# New insights into early MIS 5 lithic technological behavior in the Levant: Nesher Ramla, Israel as a case study

**DOI:** 10.1371/journal.pone.0231109

**Published:** 2020-04-03

**Authors:** Marion Prévost, Yossi Zaidner

**Affiliations:** Institute of Archaeology, The Hebrew University of Jerusalem, Mount Scopus, Jerusalem, Israel; Max Planck Institute for the Science of Human History, GERMANY

## Abstract

Interpreting human behavioral patterns during the Middle Paleolithic in the Levant is crucial for better understanding the dispersals and evolution of *Homo sapiens* and their possible interactions with other hominin groups. Here, we reconstruct the technological behavior, focusing on the centripetal Levallois method at Nesher Ramla karst sinkhole, Israel. Nesher Ramla karst sinkhole is dated to the Marine Isotope stages (MIS) 6 and 5 and represents one of the oldest occurrences of the centripetal Levallois reduction strategy in the Near East. The Levallois centripetal technology is often seen as a marker of human dispersals and adaptations in the Middle Paleolithic/Middle Stone Age of Africa and the Near East. This technology is documented in East African sites as early as 300 kya and in the Levant as early as 130 kya. However, the degree of similarity between African and Levantine centripetal technology and whether it originates from the same source remain under debate. In this paper, we focus on describing the lithic organization at Unit III of Nesher Ramla (dated to MIS 5), which is dominated by the centripetal Levallois method in association with other reduction sequences. Both preferential and recurrent centripetal Levallois modes were used at the site to produce oval and rectangular flakes. Other minor reduction sequences include unidirectional convergent method for Levallois points production and a specific method for the manufacture of naturally backed knives. The lithic data from Unit III of Nesher Ramla is further used in inter-site comparisons suggesting that the mid-Middle Paleolithic sites in the Near East possess common technological characteristics, especially the use of the centripetal Levallois method as predominant reduction strategy. This trend differs from what is usually observed in Africa and Europe, where the centripetal Levallois method is modestly represented during MIS 5 and is accompanied by other, more dominant, reduction strategies.

## Introduction

One of the most debated research topics in the prehistoric archaeology and evolutionary biology is the timing and various routes of *Homo sapiens* migrations out of Africa during the late Middle and early Late Pleistocene. The Levant plays a major role in this debate due to its location on the crossroads between Africa and Eurasia and because of the remains of modern humans and Neanderthals that were found in several caves and open-air sites in the region. Lithic artefacts are the most common cultural remains that are used as a proxy to characterize variability in human behaviors and to assess hominin dispersal patterns. On the basis of lithic record, the Levantine Middle Paleolithic (MP; ca 250–40 kya; [1–5]) was divided into the Early MP (EMP), mid-MP, and late MP (LMP), or according to the tripartite Tabun model in Tabun D, C and B [6,7]. The EMP (dated to ca 250–150 kya) is characterized by the emergence of unidirectional convergent Levallois technology alongside blade production by laminar methods [8–12]. Until recently, the makers of the EMP assemblages were unknown; however, the recent discovery of a human mandible in the EMP layer of Misliya Cave suggests that this assemblage was associated with *H*. *sapiens* [13].

The following mid-MP period, ~150-70/75 kya is characterized by the production of oval or rectangular Levallois flakes produced by the centripetal Levallois method [6,7]. The mid-MP remains poorly understood due to the small number of documented sites [14]. To date, the only mid-MP lithic data set described in detail derives from Qafzeh Cave [14]. Skhul Cave, Tabun layer C as well as Naamé, Nahr Ibrahim, and Ras El-Kelb in Lebanon are the other sites that were mentioned to represent this period [15–21]. The centripetal assemblages at Qafzeh and Skhul Caves are associated with the remains of *H*. *sapiens* [20,22–25]. The rapid accumulation of archaeological evidence from the northeastern Africa, the Arabian Peninsula, and the Levant suggests that early MIS 5 dispersals associated with the centripetal Levallois technology were a well-established phenomenon [26–32]. Despite these research advances, the degree of similarity between northeast African, Arabian and Levantine centripetal assemblages and whether they all originate from the same source remain under debate.

The paleoanthropological evidence suggests that MIS 5 human population in the Near East possibly represents one or several expansions of *H*. *sapiens* out of Africa [14,20,22,29,31,33,34]. In line with the paleoanthropological evidence, recent paleoenvironmental studies, including speleothem deposition, climatic modeling, and paleohydrology reconstructions in the Levant and Arabia have demonstrated that periods within MIS 5 were more humid, with an increase in temperatures and rainfall, which may have created “climatic windows of opportunity” for the hominid groups leaving Africa [35–43]. Some scholars take these paleoanthropological and paleoenvironmental studies as a support to African origin for the centripetal Levallois technology, that arrived to the Near East with the dispersals of *H*. *sapiens* during the MIS 5 [29,44,45]. On the contrary, the 'regional continuity' model suggests a continuous occupation of the Levant and advocates a local EMP origin for the subsequent centripetal industries of Qafzeh and Skhul [14,46]. However, given the small number of well-dated primary-context assemblages and the paucity of detailed technological studies, this question remains open and the models largely untested.

The recently excavated open-air site of Nesher Ramla karst sinkhole, in central Israel, is one of the few sites that are correlated to the mid-MP in the Near East [47–49]. Nesher Ramla site has yielded one of the largest lithic datasets dated to late MIS6 and MIS5, including ca 80 000 lithic artifacts larger than 2 cm, which contributes a significant information to the questions outlined above. The present study encompasses the lithic assemblage retrieved from Unit III of the site, which accumulated during the first half of MIS 5. The aim of this paper is to reconstruct the technological behavior of Nesher Ramla’s inhabitants. The lithic data are further used in inter-site comparisons with sites from the Levant and neighboring regions for a better understanding of the centripetal Levallois phenomenon. Our study further supports the notion that a centripetal Levallois technology dominated the mid-MP period in the Near East.

## The site

**Fig 1:**
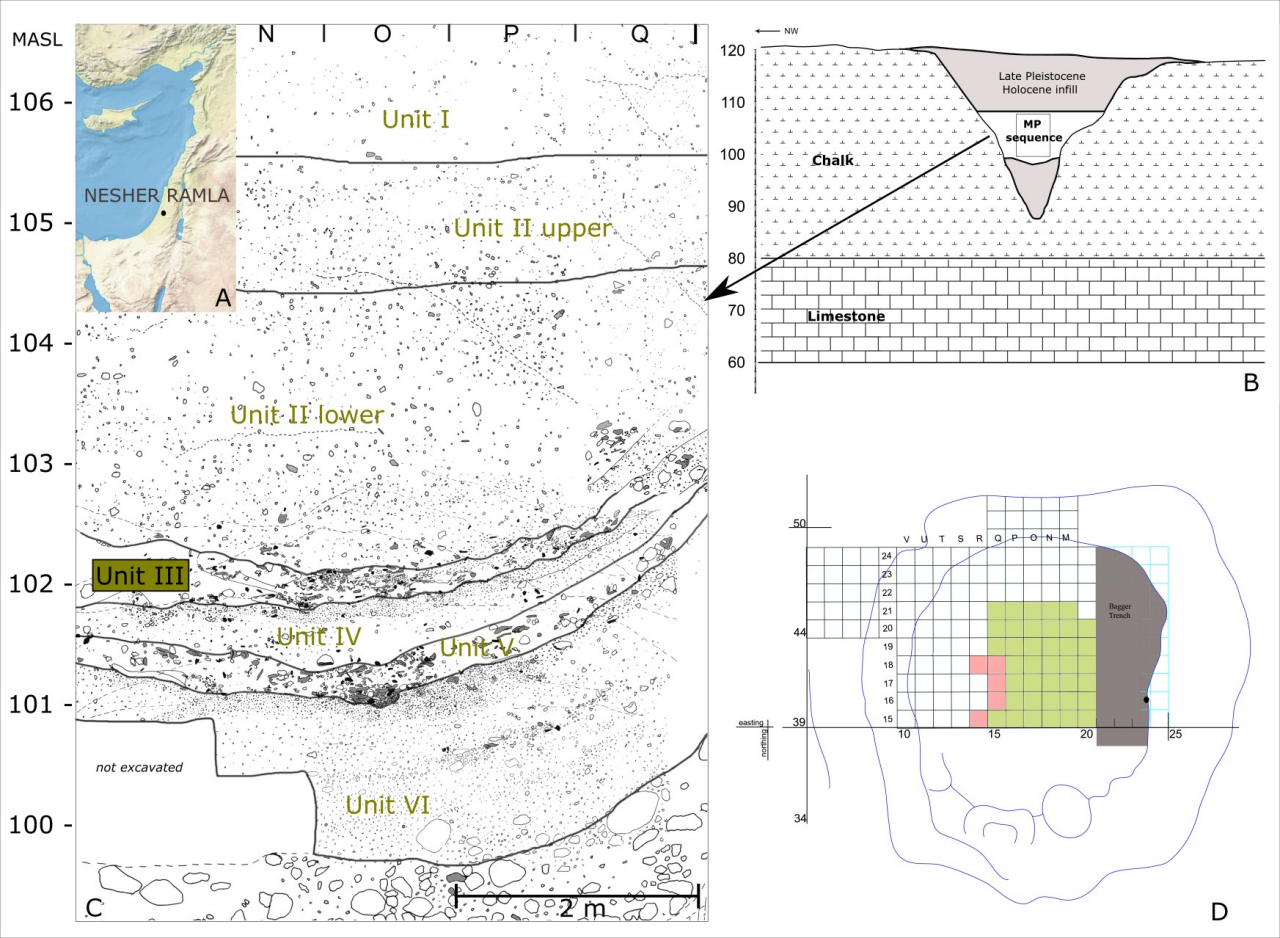
Location and stratigraphy of the Nesher Ramla site. A: The geographical location of the site (map modified after Natural Earth, public domain). B: The karst sinkhole profile with the position of the archaeological sequence. C: The stratigraphic sequence. D: The plan of the excavated area of Unit III; the green squares are those that were analyzed during this study and the pink squares are those that were excluded.

Located in central Israel, on the western slopes of the Judean Mountains, the site of Nesher Ramla was discovered following quarrying activities by the Nesher cement factory, in a deep depression formed within the chalk bedrock (Israel Antiquities Authority Permits B355/2010, B368/2011). The sagging and deforming bedrock into a deep undersurface karst void created a funnel shape depression (or sinkhole) that acted as a trap for sediments drifting from the slopes [47,48,50]. The sinkhole was completely filled with sediments and the eight-meter-thick MP layers were found in the middle of the sedimentary sequence (Fig 1). The sediments are homogeneous and composed of brown clay rich in gravel. The fine-grained sediments derived from the erosion of the surrounding soil, whereas gravels are composed of fragments of calcrete crust that developed on the chalky bedrock (also called Nari; [48]). Geoarchaeological and archaeological differences allowed the division of the archaeological sequence into six stratigraphic units (I-VI), with some internal subdivision [48,51,52]. Even though the lithic artifacts and faunal remains occur throughout the archaeological sequence, the artifact density varies, providing evidence for dynamic variation in the human occupations [48,50]. Units III and V appear to be similar, both presenting a high density of knapped flint artifacts and percussions tools (i.e. hammerstones and anvils), while units I and II show higher variation in density of the remains [48]. Several dating methods were used to date the archaeological sequence: TL, OSL, and ESR [48,49]. Six preliminary single-grain samples of OSL provided an age estimation between 160±8 and 78±6 ka. Additionally, burnt flint from Units II, III, and V were tested by TL; the preliminary results indicate an age between 117 and 185 ka [49]. Thus, the site is assigned to the end of MIS 6 and the beginning of MIS 5.

**Fig 2:**
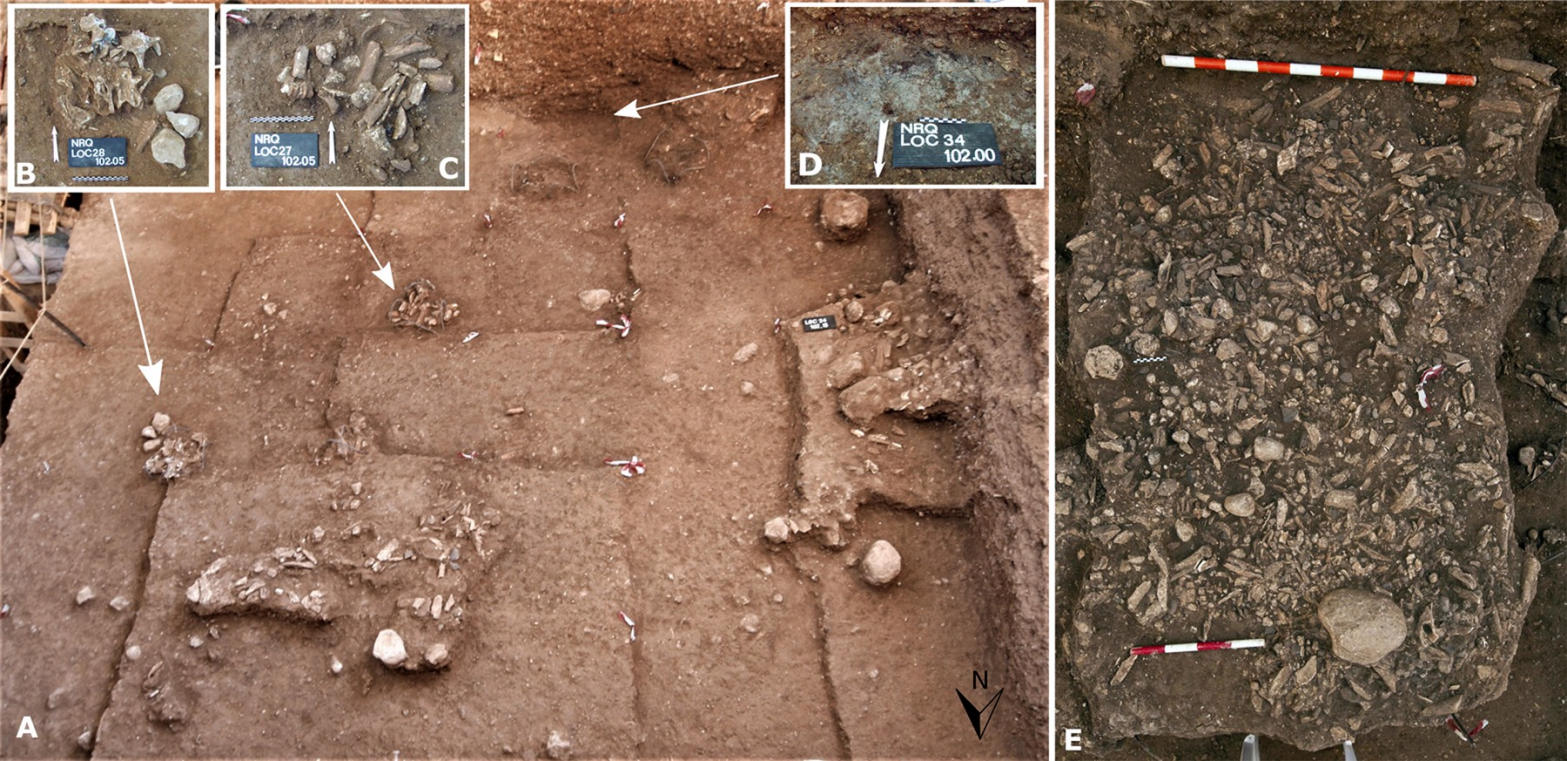
Examples of anthropogenic features found in Unit III. A: The surface of Unit III exhibiting some of the anthropogenic features. B, C: The circular accumulations of stones, bones, and flint artifacts. D: Locus 34 is a concentration of ashes, black sediments, and burnt bones. E: Locus 39 is a dense accumulation of fragmented bones, flint artifacts, and manuports.

Unit III is a distinct horizon about 40 cm thick that is located in the middle of the MP sequence [51]. Geoarchaeological analyses indicate a complex microstructure resulting from two depositional mechanisms: anthropogenic and geogenic/biogenic [48,51,53]. Unit III is one of the richest archaeological layers at Nesher Ramla and is characterized by the presence of numerous well-defined anthropogenic features which are scarce within the upper units I and II and lower units IV and VI. These features differ in size and shape; they contain bones, lithic artifacts, manuports, and ground stone tools in various quantities. Some of these features are circular in shape and resemble small “heaps” (Fig 2); others are large concentrations that extend over one meter. These features are spatially and horizontally clearly defined and contrast with the surrounding sediments. Additionally, different types of combustion features with well-preserved ashes and charcoal were identified (Fig 2D). Thin layers of blackened soil overlaid by ash and charcoal were interpreted as *in situ* hearths, whereas a 20 cm-thick massive layer of redeposited mixed ash was interpreted as resulting from hearth rake-out activities [51]. The different types of anthropogenic accumulation and combustion features may represent either distinct temporal events (single events) or several on-site visits by the same or different hominid group. In any case, the well-defined stratigraphic boundaries indicate the integrity of the lithic assemblage and make Unit III an excellent case study for analyzing human technical behavior on a restricted temporal and spatial scale.

## Materials and methods

A detailed technological and typological analysis of the entire lithic assemblage (>2 cm) of Unit III, excavated during the 2011 and 2012 field seasons was conducted. The technological analysis was conducted using a “*chaîne opératoire*” approach in an attempt to reconstruct the technological behavior, from the acquisition of the raw materials to the discard of the knapped artifacts [54,55]. The detailed attribute analysis combines qualitative and quantitative attributes to better characterize and quantify the different reduction sequences, as well as their extent and the different aspects of the raw material economy [14,56]. The typological classification follows Bordes' type-list [57]. A few categories were added according to specific tool types retrieved at Nesher Ramla (i.e., tools with a lateral tranchet blow). Non-retouched Levallois flakes and naturally backed knives (NBKs) were classified within débitage production and not within tools.

Emphasis was placed on describing the Levallois reduction strategy, since it is the dominant system used at the site and because of its importance in the debate on the Levantine MP chrono-cultural framework. Identification of the Levallois concept is based on criteria proposed by Boëda [58–60]. The general scar structure of the core’s débitage surface allows the identification of different production modes: preferential and recurrent as well as various methods. The preferential mode corresponds to the production of a single, invasive, and centered end-product (predetermined product), after a careful preparation of the flaking and striking surfaces. Thus, this mode generates a high number of preparation products (predetermining products). The preferential Levallois flakes bear exclusively predetermining scars on their dorsal surface and, are expected to be large and symmetrical with a point of percussion at the center of the striking platform [61]. Using the preferential mode of reduction, the knappers aimed at removing the largest flake possible, which is not an economical flaking mode in terms of flake productivity, since one core will only produce a limited number of end-products [54,62]. On the other hand, the preferential mode results in flakes with the longest working edge possible and thus is preferable in terms of edge efficiency [63]. Conversely, the recurrent mode aims at producing a series of predetermined Levallois flakes of more diverse sizes, which can act simultaneously as preparation and end-products [59].

Within the Levallois system, it is possible to distinguish between the preparation and exploitation phases by analyzing the core’s scar characteristics as well as their position, orientation, order of removal, and invasiveness. On the Levallois cores, the predetermining flakes (i.e., the preparation flakes) were usually aimed at shaping the core débitage surface to obtain suitable convexities for better control over the shape of the final products (i.e., the predetermined flakes). The negatives of the predetermining flakes appear secant to the plane of intersection and are usually less invasive than the predetermined ones. Experimental studies for the production of preferential Levallois flakes coupled with quantitative analyses have shown that Levallois flakes (i.e., the end-products) can be distinguished from other flakes produced during their manufacture (i.e., the by-products), by their morphology and metrical features [62,63]. Compared to the preferential mode of reduction, the Levallois recurrent system involves preparation and exploitation phases that are more tenuous to recognize. Therefore, the Levallois flakes produced by this mode appear to be less standardized in terms of morphology [61] and more difficult to be identified. All of these characteristics have allowed researchers to identify a large diversity of methods of exploitation and preparation (e.g., unidirectional parallel and convergent, centripetal, and bidirectional), which can occur in different combinations within a single reduction sequence (e.g., centripetal preparation with centripetal exploitation or centripetal preparation with unidirectional exploitation; S1 Fig).

Flint from several sources were used at Nesher Ramla. The flint types were classified into Mishash, Eocene, and “indeterminate” flint. This classification was carried out with the naked eye, based on the colors, textures, and presence of fossils. The Mishash flint, part of the Campanian Mishash Formation, is available in abundance in the vicinity of Nesher Ramla (15 km). The “indeterminate” and Eocene flints most probably originated from sources located at a distance of more than 15 km from the site (R. Ekshtain, pers. comm.).

In addition, refitting analysis was conducted. Because of the dominance of Mishash flint artifacts that bear similar visual and textural characteristics, the refitting study was only conducted for the “indeterminate” and Eocene flint types, which represent around 30% of the total assemblage. These types of flint have distinctive visual characteristics (e.g., colors, textures, and cortex types) allowing their grouping by Raw Material Units [64]. For tools with lateral tranchet blows and spalls, the refitting study included all the raw material types.

For the statistical analysis we used the Pearson Chi-Square parametric test to compare qualitative variables and, the Mann-Whitney non-parametric test for comparing quantitative variables (p value = 0.05). These statistical analyses were conducted using PAST, version 3.22 software [65].

The studied sample originates from an area of 39 square meters; this covers almost the entire excavated area of Unit III (Fig 1D). Five squares located at the limit of the excavation area exhibited some stratigraphic disturbances and were not included in the studied sample (Fig 1D). The lithic assemblage is composed of 11 084 artifacts larger than 2 cm, 15 632 micro-artifacts smaller than 2 cm, 110 knapped limestone artifacts (S1 Table), and a large assemblage of manuports and percussion tools (Table 1 and S2 Table). The large lithic sample represents all stages of the reduction sequences, as identified by cores and distinctive technical pieces.

**Table 1:** Composition of the studied lithic (flint) assemblage.

		N	% (on total assemblage >2cm)	Transformed into tools (N)	% of retouched tools
**DEBITAGE**					
Flake		4317	38.9%	395	32.2%
Blade		213	1.9%	16	1.3%
Kombewa flake		196	1.8%	19	1.5%
Core trimming element		1053	9.5%	146	11.9%
Levallois flake		1096	9.9%	292	23.8%
Levallois blade		30	0.3%	14	1.1%
Levallois point		73	0.7%	21	1.7%
Cortical flake 25–75%		760	6.9%	82	6.7%
Cortical flake 76–99%		1018	9.2%	57	4.6%
Entame		92	0.8%	0	0.0%
NBK		1002	9.0%	154	12.6%
Retouched tranchet blow spall		216	1.9%	0	0.0%
Tranchet blow spall		85	0.8%	7	0.6%
Nahr Ibrahim piece		82	0.7%	10	0.8%
Chunk		417	3.8%	1	0.1%
*sub-total débitage products*		**10 650**	96.1%		
**CORE**					
Levallois core (LEVC)		155	1.4%	4	0.3%
NBK production core (NBKC)		39	0.4%	0	0.0%
Hierarchical surface core (HSC)		105	0.9%	2	0.2%
Multi-surface core		20	0.2%	0	0.0%
Opposed platform core		2	0.0%	0	0.0%
Single platform core		12	0.1%	0	0.0%
Globular core		2	0.0%	0	0.0%
Core-on-flake		71	0.6%	6	0.5%
Core fragment		23	0.2%	0	0.0%
Tested nodule		5	0.0%	0	0.0%
*Sub-total cores*		**434**	3.9%		
**RETOUCHED TOOLS**		** **	** **	**1226**	
**TOTAL (>2cm)**		**11 084**	41%		
**MICRO-ARTEFACTS (<2cm)**		15 632	58%		
**LIMESTONE DEBITAGE**		110	0.4%		
**MANUPORT**		323	1%		
**TOTAL**		**27 149**	100%		

## Results

### Raw material exploitation

**Fig 3:**
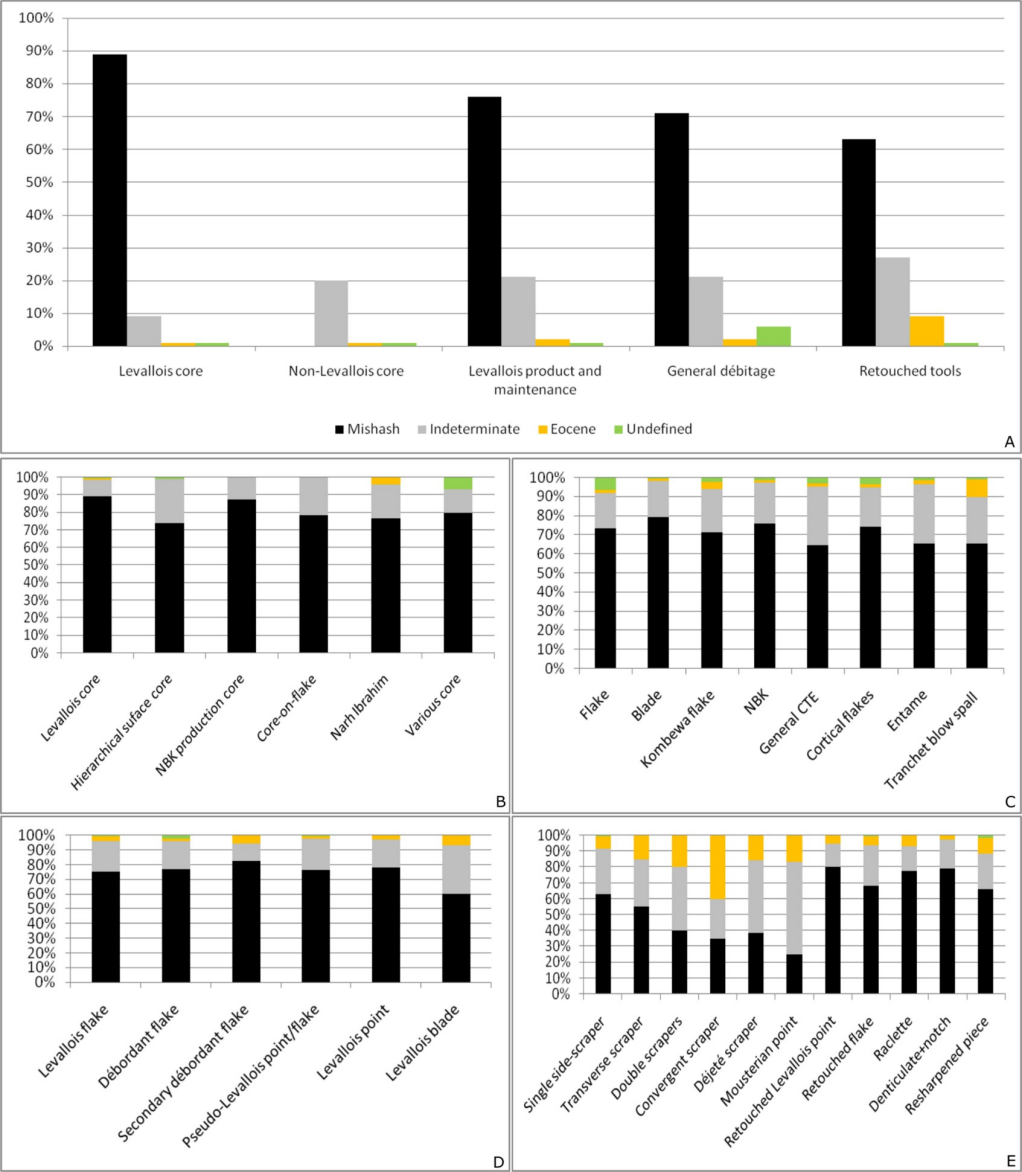
Raw material representation of the Unit III lithic assemblage. A: General assemblage. B: Core types. C: Non-Levallois débitage categories. D: Levallois end- and by- products. E: Retouched tools.

Local Campanian flint of Mishash Formation is the most common raw material at Nesher Ramla (71%), whereas flint of indeterminate sources (31%) and flint of Eocene age (3%) are less common. Analysis of the cortex type revealed that flint from both primary and secondary sources were exploited. The provenience of the majority of the Eocene and the “indeterminate” flint types is still unknown; they were probably transported from longer distances. Both Mishash and “indeterminate” flint, appear in similar frequencies within the different artifact groups (Fig 3). Differences occur mainly within the Eocene flint, with an overrepresentation of retouched tools and Levallois products.

In addition, a small number of limestone artifacts were identified (1%). The limestone was used sporadically, as indicated by a small number of débitage components (S1 Table); therefore, this assemblage is not included in our detailed technological analysis.

### Technological analysis

The débitage products of Unit III are represented by different types of blanks ranging from cortical elements to retouched tools. This implies that knapping activities took place on site. Cortical elements, divided according to the amount of cortex on the dorsal face (Table 1), are represented in high frequencies (16%). The preservation of the lithic assemblage is good; the flint artifacts are fresh, without signs of weathering or rolling. It is interesting to note that 6.4% of the lithic sample exhibit a double patina suggesting that some recycling was employed.

### The Levallois reduction system

#### The cores

**Fig 4:**
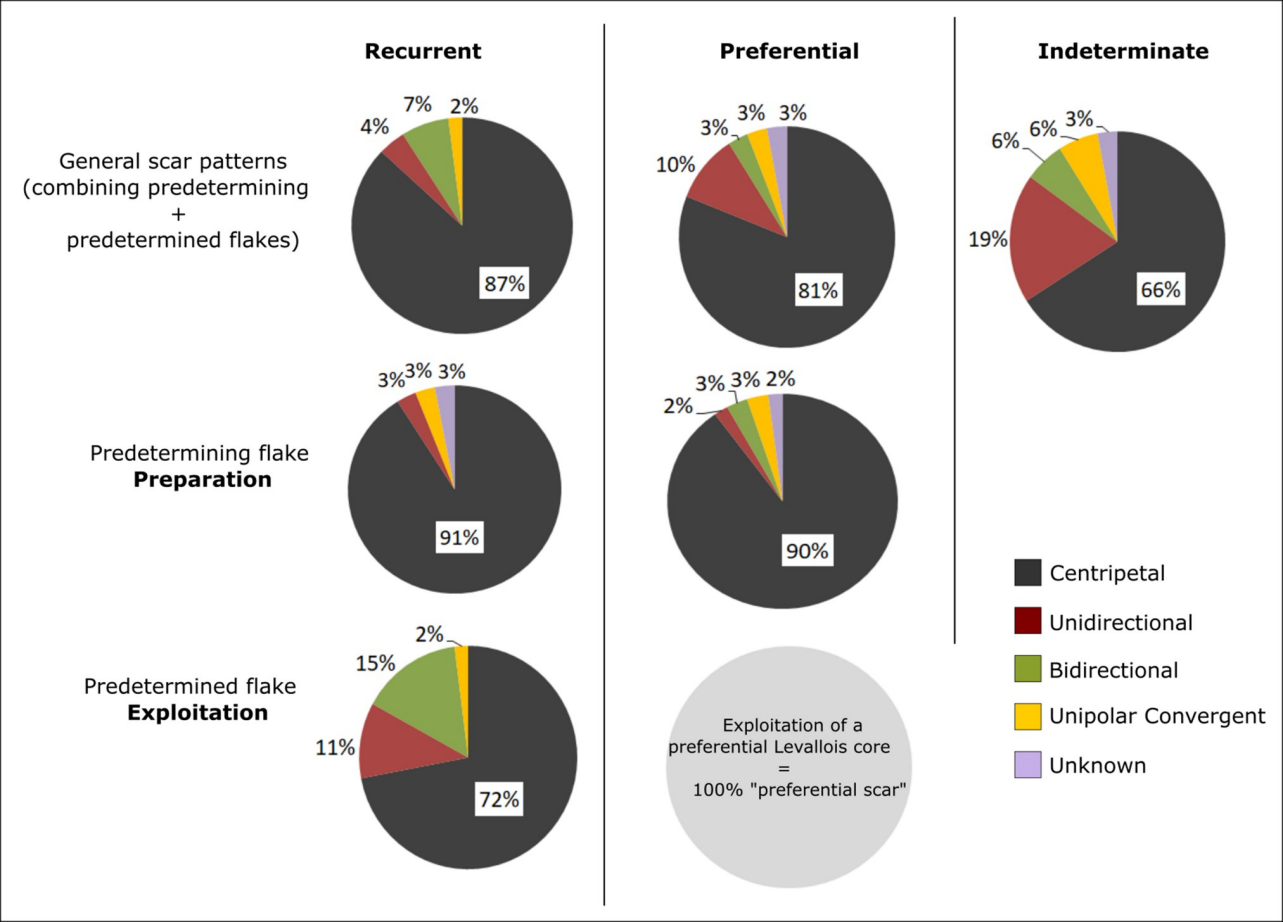
Levallois cores scar patterns. Directions of the scar patterns on Levallois cores according to the modes and methods of preparation and exploitation.

The local Mishash flint dominates the Levallois cores’ assemblage (89%). Cortex remained on 88% of the cores, which, in some cases, allowed us to identify the source of the raw materials. According to comparisons made with nodules and pebbles sampled during the raw material surveys, 43% of cores were made of nodules from primary sources (i.e. irregular and angular nodules) and 6% were made of pebbles from secondary sources (i.e. round and smooth cortical texture). Six Levallois cores were made on flake. The Levallois cores are thin, often until a point that further reduction is impossible and thus, they are considered as exhausted (56%). The scar organization on the dorsal face of the cores indicates that both preferential and recurrent reduction modes were used at Nesher Ramla (Fig 4 and S1 Fig). The preferential Levallois cores (45.2%) are more abundant than the recurrent ones (34.8%; Table 2). A series of Levallois cores was identified as “indeterminate” because the difference between the predetermining and predetermined scars was ambiguous and this, did not allow them to be identified either as preferential or recurrent. The preferential and recurrent Levallois cores have similar sizes (Table 2), whereas indeterminate Levallois cores are smaller and thicker. The average length of the longest scar on the flaking surface appears to be longer on the preferential cores than on the recurrent ones (Mann-Whitney *U* = 1180; z = 2.1292; p = 0.033).

**Table 2:** The cores’ metric data.

		Levallois core	*Preferential*	*Recurrent*	*Indeterminate*	Hierarchical surface core	NBK production core	Core-on-flake	Various cores	total
	
	n	155	70	54	31	105	39	71	59	429
	%	30.3%	45.2%	34.8%	20.0%	24.5%	9.1%	16.6%	13.8%	
Length	Mean	46.89	47.28	47.64	42.78	45.6	47.44	46.23	43.54	
	Sd	9.47	9.6	9.64	7.66	9	9.14	9.9	9	
	Max	78.9	78.9	78.8	59.8	79.6	0	43.8	0	
	Min	30.6	30.6	31.8	31	29.8	0	37	0	
Width	Mean	40.73	40.9	41.38	37.92	38.72	39.63	37.2	34.98	
	Sd	8.66	8.6	8.95	7.9	9.01	7.49	8.96	8.32	
	Max	71.8	62.6	71.8	55.7	77.8	0	37,9	0	
	Min	23.5	23.5	25.9	27.7	24.6	0	29.1	0	
Thickness	Mean	20.88	20.75	20.83	21.61	20.49	20.89	15.71	24.42	
	Sd	6.64	6.79	6.56	6.68	7.06	5.1	4.65	9.07	
	Max	46.4	46.4	41.3	40.2	52.1	0	20.1	0	
	Min	9.7	10.1	9.7	14.1	9.6	0	11.4	0	
Length of the longest scar	Mean	30.3	31.78	28.25	30.77	29.19	38.93	25.9	27.56	
	Sd	9.3	8.95	8.85	11.4	10	9.61	10.02	10.11	
	Max	56.5	51.7	56.5	43.9	53.7	0	29.3	0	
	Min	13.5	13.5	14	13.5	11.9	0	13.4	0	

The striking surface of the Levallois cores shows a high investment of preparation by faceting (95% facetted and 3% dihedral striking surfaces). The faceting occurs on more than half of the circumference of the striking platform.

**Fig 5:**
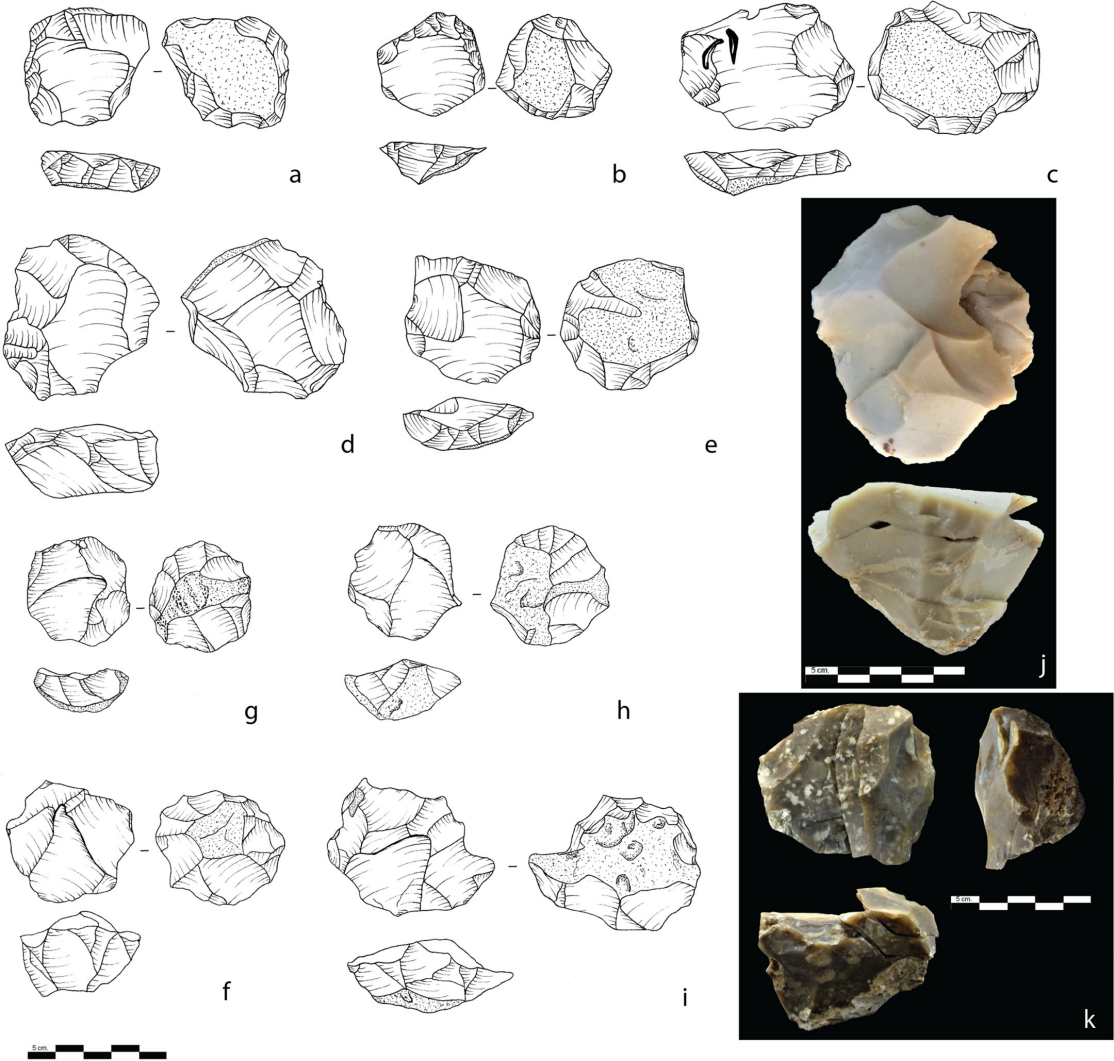
Levallois cores. a, b, c, d, e: Preferential Levallois cores; f, g, h, i: Recurrent Levallois cores; j: A refit of a Levallois core, a Levallois flake, and a flaking surface rectification flake; k: A refit of a Levallois core with 2 superimposed *débordant* flakes.

In order to distinguish between the preparation and the production stages of the Levallois reduction system, we attempted to differentiate between predetermining and predetermined negative scars. The Levallois cores exhibit an average of 6.22 scars (≥ 5 mm) on their flaking surface and their general organization indicates that the centripetal system dominates (87% for the recurrent mode and 81% for the preferential mode) (Figs 4 and 5). The majority of the indeterminate Levallois cores also exhibit a centripetal flaking pattern (65%). Unidirectional, bidirectional, and convergent scar patterns appear sporadically for both Levallois reduction modes. Predetermining flake scars, when identifiable, were found to be mostly centripetal (90%) for both recurrent and preferential Levallois cores. This indicates that the dominant and probably the only method for débitage surface and convexity preparation was the centripetal. Predetermined flake scars, although exhibiting a slightly greater scar pattern variability, still indicate that the centripetal method also dominated the production stage (72%).

The preferential Levallois cores with last hinged flake removal (n = 9), are larger than the other preferential Levallois cores (52.6x45.3x27.6 mm; SD: 14.6; 11.7; 11.3 and 46.3x40 x19.5 mm; SD: 8.4; 7.8; 5 respectively), suggesting that the hinge was the reason for their discard. On only 6% (n = 9) of the Levallois cores, “secondary” flakes that correspond to a new predetermining stage of flaking were identified. They appear secant to the plane of intersection and are organized centripetally around the periphery of the flaking surface. A study of the core’s reduction history indicates that 23% of the Levallois cores exhibit one or several additional flakes that were removed after a last series of Levallois removals (S2 Fig). These last-stage removals reflect an opportunistic knapping behavior applied to maximize the core exploitation. This opportunistic behavior was described as a “profit situation” (“*méthode conjecturale*”) by Boëda and colleagues [58]. The majority of the last-stage opportunistic flakes (59%) and the new predetermining flakes (67%) occur on preferential Levallois cores.

#### The core trimming elements

**Fig 6:**
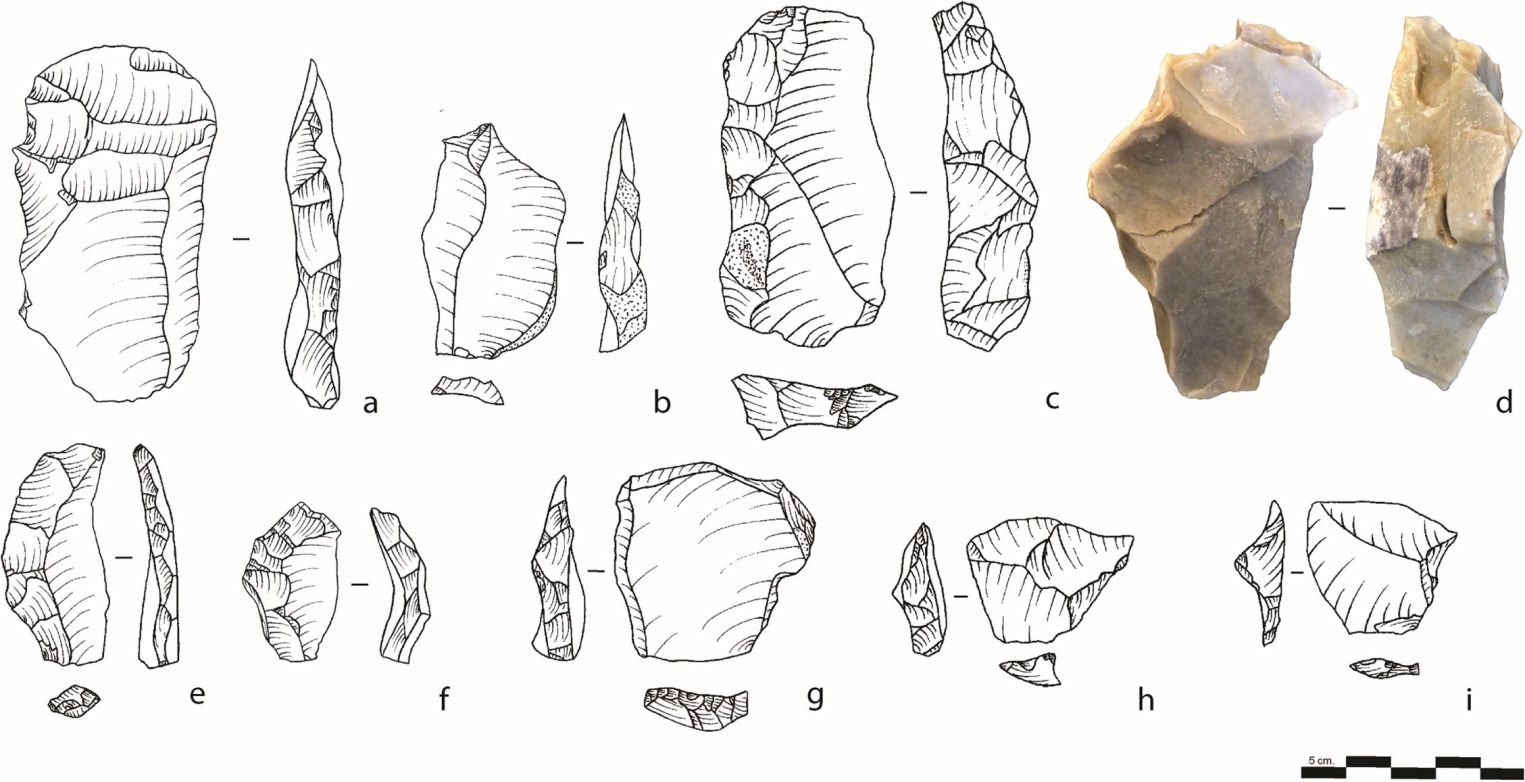
Core trimming elements. a, b, c: “Primary” *débordant* flake; d: A refitting of two “primary” *débordant* flakes; e, f: “Secondary” *débordant* flakes; g: Pseudo-Levallois flake; h, i: Pseudo-Levallois points.

Several types of core management pieces associated with the Levallois reduction sequences were identified (Table 3). The large quantity of core management pieces in the sample indicates that knapping sessions and re-organization of the Levallois core convexities was an important part of the knapping activities at the site. The largest category is represented by the *débordant* flakes, which is aimed to modify the lateral edges of the Levallois cores in order to prepare new convexities. These characteristic flakes possess a back that bears residue scars from the preparation of striking platforms. The centripetal scar pattern is the most frequent and the striking platforms are mostly facetted (Table 4). Following the definition by Geneste [54], two types of *débordant* flakes were recognized: (1) “primary” *débordant* flake (i.e., Éclat *débordant primaire*) characterized by centripetal, bidirectional, or unidirectional scar negatives resulting from a previous recurrent reduction sequence, (2) “secondary” *débordant* flake (i.e., Éclat *débordant* “*secondaire”*), characterized by a negative scar of a preferential Levallois removal (Fig 6). Identifying these sub-types of *débordant* flakes provides better understanding of how often and at which stage of the reduction, the recurrent and preferential modes were used. The “secondary” *débordant* flakes, removed from preferential Levallois cores, bear mostly centripetal (and “horse shoe”) scar patterns and are significantly larger than the “primary” *débordant* flakes (Table 5; Mann-Whitney *U* = 1942; z = 3.0478; p = 0.0023), indicating that a series of preferential Levallois flakes was produced at an early stage of Levallois reduction.

**Table 3:** Amount and types of core trimming elements (including blanks for tools).

Core management elements	n	%
General CTE	136	13%
*Dédordant flake*	452	43%
Primary *débordant* flake	*416*	*92%*
Secondary *débordant* flake	*36*	*8%*
*Pseudo Levallois flake*	225	21%
Pseudo Levallois point	26	2%
*Overshoot flake*	39	4%
*Débordant and overshoot flake*	112	11%
Flaking surface rectification flake	19	2%
Striking platform rectification flake	44	4%
	1053	100

**Table 4:** Scar patterns and striking platform types according to selected blank categories (including retouched tools).

		Flake		Levallois flake		Levallois point		NBK		Cortical flake (25–75%)		*Débordant* flake		Pseudo-Levallois point/flake	
		n	%	n	%	n	%	n	%	n	%	n	%	n	%
Scar patterns^a^	Centripetal (and orthogonal)	694	32.7%	543	67.0%	20	27.4%	142	18.2%	62	13.2%	303	64.1%	145	66.8%
	Unidirectional	615	29.0%	79	9.8%	5	6.8%	461	59.1%	267	56.9%	64	13.5%	12	5.5%
	Bidirectional	67	3.2%	29	3.6%	2	2.7%	66	8.5%	10	2.1%	21	4.4%	6	2.8%
	Convergent	70	3.3%	64	7.9%	45	61.6%	9	1.2%	5	1.1%	2	0.4%	12	5.5%
	Indeterminate	675	31.8%	95	11.7%	1	1.4%	102	13.1%	125	26.7%	83	17.5%	42	19.4%
*Total*		2121	100%	810	100%	73	100%	780	100%	469	100%	473	100%	217	100%
Striking platform^b^	Facetted	820	29.4%	807	79.3%	52	75.4%	422	46.4%	128	23.1%	289	53.6%	129	52.0%
	Chapeau de gendarme	0	0.0%	33	3.2%	8	11.6%	2	0.2%	0	0.0%	1	0.2%	0	0.0%
	Dihedral	429	15.4%	90	8.8%	7	10.1%	84	9.2%	62	11.2%	50	9.3%	26	10.5%
	Plain	1157	41.5%	50	4.9%	0	0.0%	299	32.9%	262	47.2%	147	27.3%	76	30.6%
	Cortical	109	3.9%	1	0.2%	0	0.0%	52	5.7%	54	9.7%	14	2.6%	7	2.8%
	Removed	94	3.4%	24	2.4%	0	0.0%	26	2.9%	13	2.3%	20	3.7%	2	0.8%
	Punctiform	41	1.5%	0	0.0%	0	0.0%	3	0.3%	8	1.4%	7	1.3%	0	0.0%
	Crushed	114	4.1%	8	0.8%	1	1.4%	14	1.5%	19	3.4%	6	1.1%	0	0.0%
	Indeterminate	26	0.9%	5	0.5%	1	1.4%	7	0.8%	9	1.6%	5	0.9%	8	3.2%
*Total*		2790	100%	1018	100%	69	100%	909	100%	555	100%	539	100%	248	100%

^**a**^ Includes only the complete pieces

^**b**^ Includes only the artifacts with the remaining proximal parts.

**Table 5:** General metrics data of selective types of blanks.

		Length (mm)		Width (mm)		Thickness (mm)	
		Mean	SD	Mean	SD	Mean	SD
Débitage	Flake (n = 2121)	30.43	12.45	25.09	9.58	5.7	3.41
	Flake" tool" (n = 196)	46.19	12.33	32.97	8.16	9.78	3.31
	NBK "flake" (n = 671)	46.08	11.17	28.05	7.38	8.85	3.93
	NBK "blade" (n = 129)	52.9	12.57	22.5	6.87	8.14	3.31
	Kombewa flake (n = 109)	37.33	13.29	28.14	9.30	6.15	3.89
	Cortical elements (n = 1073)	39.01	14.71	28.97	10.20	8.59	4.37
	Nahr Ibrahim piece	46.11	9.19	33.38	8.33	11.8	3.49
Levallois end-product and *débordant* flakes	*Débordant* flake (n = 452)	43.56	12.67	26.68	9.24	8.35	3.81
	Primary *débordant* flake (n = 307)	44.42	12.79	28.44	9.95	8.8	3.84
	Secondary *débordant* flake (n = 21)	53.24	12.83	26.93	9.47	8.23	3.71
	Levallois flake (n = 810)	42.64	13.38	35.05	10.62	5.88	2.66
	unidirectional (n = 75)	44.27	13.88	34.41	9.68	5.71	2.47
	bidirectional (n = 29)	50.21	14.8	37.35	9.63	7.01	2.66
	convergent (n = 64)	43.72	13.39	32.32	8.6	6.05	2.9
	centripetal and orthogonal (n = 543)	41.92	13.33	35.12	11.10	5.64	2.62
	Preferential Levallois flake (n = 153)	47.95	13.80	42	10.72	6.32	2.57
	"Recurrent" Levallois flake (n = 657)	41.38	12.97	33.52	9.92	5.8	2.68
	Levallois flake "tool" (n = 232)	49.78	12.47	39.16	9.73	7.57	2.38
	Levallois flake Mishash (n = 562)	41.31	13.13	34.37	10.37	5.58	2.60
	Levallois flake "indeterminate" flint (n = 191)	44.57	13.12	36	10.88	6.45	2.62
	Levallois flake Eocene (n = 57)	49.99	13.75	38.52	11.86	7.09	2.85
	Levallois point (n = 67)	47.45	11.52	33.25	9.32	5.6	2.09
	classical (n = 36)	44.66	8.56	31.41	7.91	5.62	2.03
	constructed (n = 31)	50.9	13.59	35.61	10.38	5.58	2.24
	Retouched Levallois point (n = 21)	49	10.07	33.43	9.59	6.15	2.16

The pseudo-Levallois flakes [57] (or *éclat débordant à dos limité* by [54]) and pseudo-Levallois points (Fig 6G, 6H and 6I) represent a large portion of the core trimming elements associated with the Levallois system (22% and 3%, respectively, Table 3), which implies a high degree of core rejuvenation and successive series of Levallois products removals. Compared to the *débordant* flakes, the pseudo-Levallois flakes and points have a sub-rectangular or triangular/sub-triangular morphology. The removal axis is not parallel to the back of the artifact as with the *débordant* flakes and, consequently, their morphological and flaking axes converge, making the distal part offset [see also 12,59].

Pseudo-Levallois points are sometimes associated with the discoid reduction sequence [66–68]. At Nesher Ramla, evidences for the use of the discoidal method, including discoidal cores are absent. Pseudo-Levallois points are interpreted as part of the Levallois centripetal system as previously suggested for other MP sites in which centripetal Levallois system was employed [61]. The removal of pseudo-Levallois flakes and points, which can be considered as an end-product in some cases [69], has a limited effect on the flaking surface and allow more precise control over the maintenance of core convexities than do the *débordant* flakes [61]. In our sample, the pseudo-Levallois flakes and points are thinner than the *débordant* flakes and exhibit centripetal (46%) and orthogonal (20%) scar patterns; except for the pseudo-Levallois points, which generally exhibit a convergent scar pattern (38%) (Table 4). This can be explained by the fact that they remove only a restricted zone of the centripetally prepared/exploited flaking surface (S3 Fig).

Another type of core management element (Table 3), is the flaking surface rectification flake, which serve at cleaning a surface covered by hinged scars or irregularities in the flint, as illustrated by a refit (Fig 5J). Only a few of these flakes were identified, either because the knappers discarded the core rather than re-shaped it (as seen in the Levallois core sample, where 11% of them exhibit hinged scars), or alternatively, the knappers re-prepared the flaking surface by removing *débordant* or pseudo-Levallois flakes. Additional types of core management elements were identified in the sample including “striking platform flakes”, which aimed at preparing the core’s striking surface.

#### The Levallois products: Flakes

The Levallois production is flake oriented (91%). The Levallois flakes (n = 1097; 9.9% of the entire assemblage) are mostly made on local Mishash flint (69%). Because their detachment follows a series of predetermining flake removals, the predetermined flakes are largely devoid of cortex and only 11% of the Levallois flakes bear up to 25% of cortical cover.

Even though different scar patterns were identified on Levallois flakes, it is clear that the centripetal method was the most frequently employed. The centripetal scar pattern dominates the assemblage (67%) (Table 4). Flakes with unidirectional, bidirectional, and convergent scar patterns were identified in low frequencies (Table 4). On average, the flakes removed using the Levallois method bear more dorsal scars and are bigger than the regular flakes (Table 5). This is particularly true for the Levallois flakes with centripetal scar pattern (a mean of 5.4 scars). The Levallois flakes exhibit a facetted striking platform in 79.3% of cases. “*Chapeau de gendarme*” striking platforms appear in low frequencies (3.2%; Table 4) and are mostly associated with centripetal Levallois flakes rather than convergent Levallois flakes.

Many Levallois flakes exhibit an irregular morphology (62%), with both lateral edges being non-symmetrical. This is possibly caused by the frequent use of the Levallois recurrent system, which commonly produces flakes of different sizes and of more diversified shapes [61]. The recurrent centripetal Levallois system is characterized by the cordal direction of knapping [66]; this makes it difficult to control the products’ shapes, which can explain the irregular and often non-symmetric shapes of the Levallois flakes.

**Fig 7:**
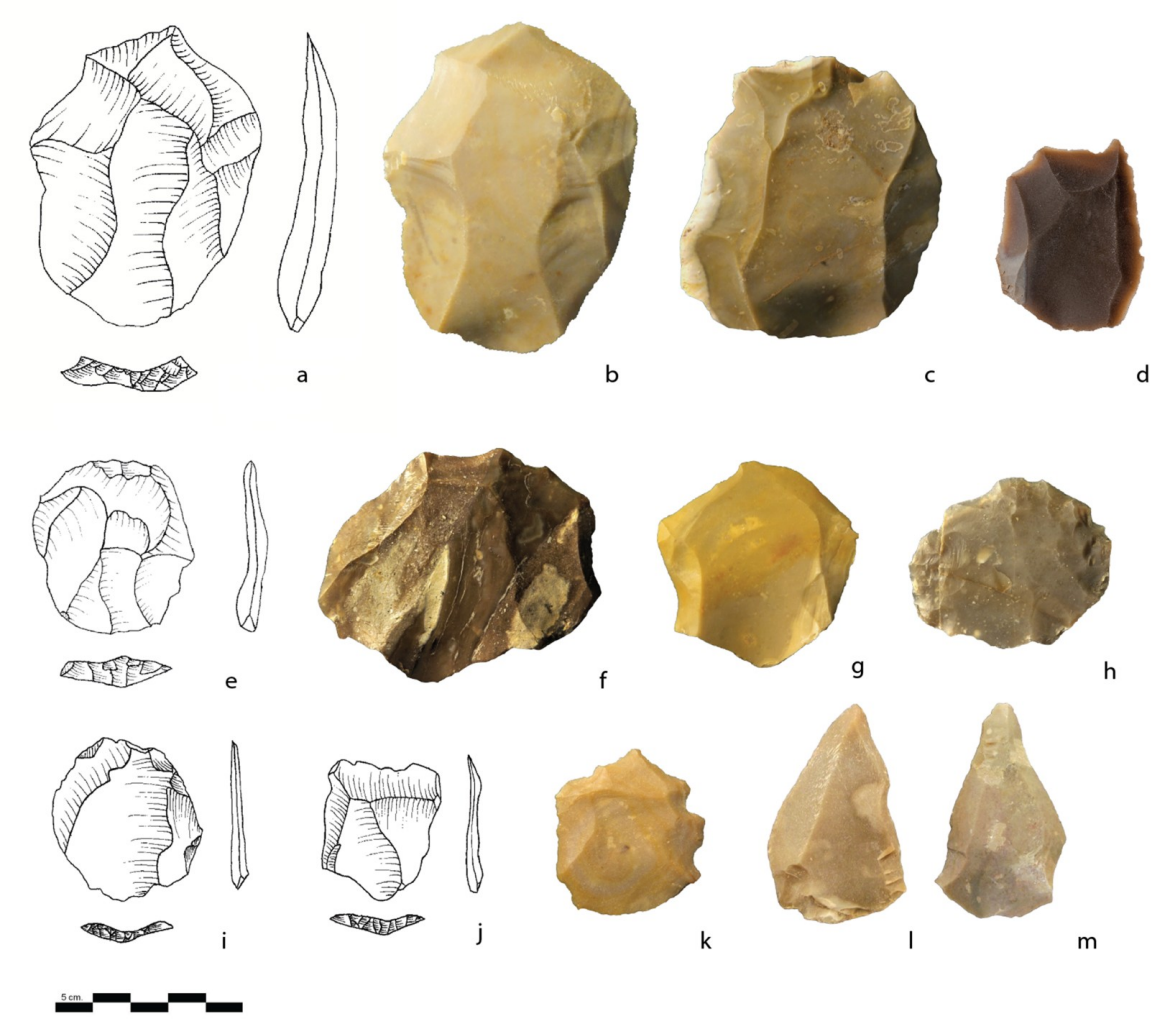
Levallois products. a-k: Levallois flakes (mostly preferential); l, m: Levallois points.

We attempted to identify preferential Levallois flakes (Fig 7), in order to quantify each Levallois mode employed at the site as well as to identify possible shifts in production mode (from preferential to recurrent and *vice versa*) during the knapping process. Using criteria provided above we have identified 200 preferential flakes which contribute 18% to the Levallois flakes assemblage. This is likely to represent a minimum number, since smaller preferential Levallois flakes or the ones less standardized in shape may have been missed. The preferential Levallois flakes are significantly larger than the recurrent Levallois flakes (Table 5; Mann-Whitney *U* = 36037; z = 5.414; *p*<0.05) and have a symmetrical sub-rectangular/oval morphology. The preferential Levallois flakes are 30–60 mm long and are characterized by an extensive preparation of the striking platforms (95% are facetted or “*chapeau de gendarme*” types).

Several observations suggest that the unidirectional parallel, convergent, and bidirectional Levallois reduction methods were used at the beginning of the reduction sequence. Levallois flakes with unidirectional parallel, convergent, and bidirectional scar patterns are significantly longer than the centripetal ones (Mann-Whitney *U* = 38743; z = 2.89; *p* = 0.0038). In addition, cortical remains (between 1% and 25%) appear mostly on unidirectional parallel, convergent, and bidirectional Levallois flakes (25%, 25%, and 21%, respectively, versus 8% on centripetal and orthogonal flakes). Thus, we suggest that Levallois flakes with unidirectional, convergent, and bidirectional scar patterns were produced at an early stage of the core reduction and that they were subsequently reduced by the centripetal method.

#### The Levallois products: Points

The Levallois point production represents a minimal component of Unit III assemblage. No Levallois cores for points were identified and the Levallois points comprised only 1% of the total assemblage (Fig 7). The Levallois point exhibit either unidirectional convergent (62%) or centripetal (27%) scar patterns. The later occur in small numbers and it is likely that they were produced unintentionally during the flake production using the recurrent centripetal Levallois method. The dorsal scar ridges indicate the presence of classical Levallois points (3 scar points) (54%) and constructed points (more than 3 scar points) (46%) [70,71]. The later type of points exhibits either unidirectional convergent or centripetal scar patterns. The order of removals on the classical Levallois points indicates that the first blow starts on either the middle or the lateral edge of the core in equal frequencies. The constructed Levallois points appear to be longer and wider than the classical Levallois points. The latter seem to be more standardized in terms of dimensions, exhibiting ranges with lower standard deviations (Table 5).

According to the symmetry of the edges, the dorsal scars and the striking platform, the Levallois points were mostly produced by the preferential unidirectional convergent Levallois reduction mode.

#### The non-Levallois flakes

Non-cortical flakes that were not assigned to specific reduction sequence contribute to 38,9% of the assemblage. These flakes exhibit a minimal preparation of the striking platform (41.5% plain and 15.4% dihedral striking platforms) and the centripetal scar pattern slightly dominates over the unidirectional parallel one (29%; Table 4). Non-cortical flakes could have been produced by simple reduction sequences from unprepared cores; however, they also could have been produced during the preparation and maintenance of the Levallois cores. Identification of the Levallois predetermined and predetermining products remains challenging, especially when the recurrent system is employed [14,72]. As it has been shown in several experimental studies, preparation flakes (“waste”), which do not exhibit characteristics of Levallois predetermining or predetermined products are produced in high frequencies at different Levallois reduction stages, depending on the original shape and size of the nodule [63,73]. In his work, Geneste [54] also showed that during the Levallois reduction sequence a large proportion of regular flakes are produced (40%). Therefore, it is clear that at least some of the non-cortical flakes in Unit III of Nesher Ramla were produced using the Levallois flaking system.

### The naturally backed knife reduction sequence

The naturally backed knives (NBKs) were sometimes described as a tool type characterized by a cutting edge opposed to a thicker natural back suitable for gripping [57]; however, more often as “core management elements” resulting from preparing the core convexities, thus having the same function as the *débordant* flakes. The production of cortically backed elements is a well-established MP phenomenon that in most cases, intended at the preparation of lateral convexities of the Levallois cores (e.g. cortical *débordant* [74]). Nevertheless, the presence of specific core type from which only NBKs were produced and the high frequency of NBKs in the assemblage suggest that, in Nesher Ramla, intentional production of NBKs was employed.

#### The cores

**Fig 8:**
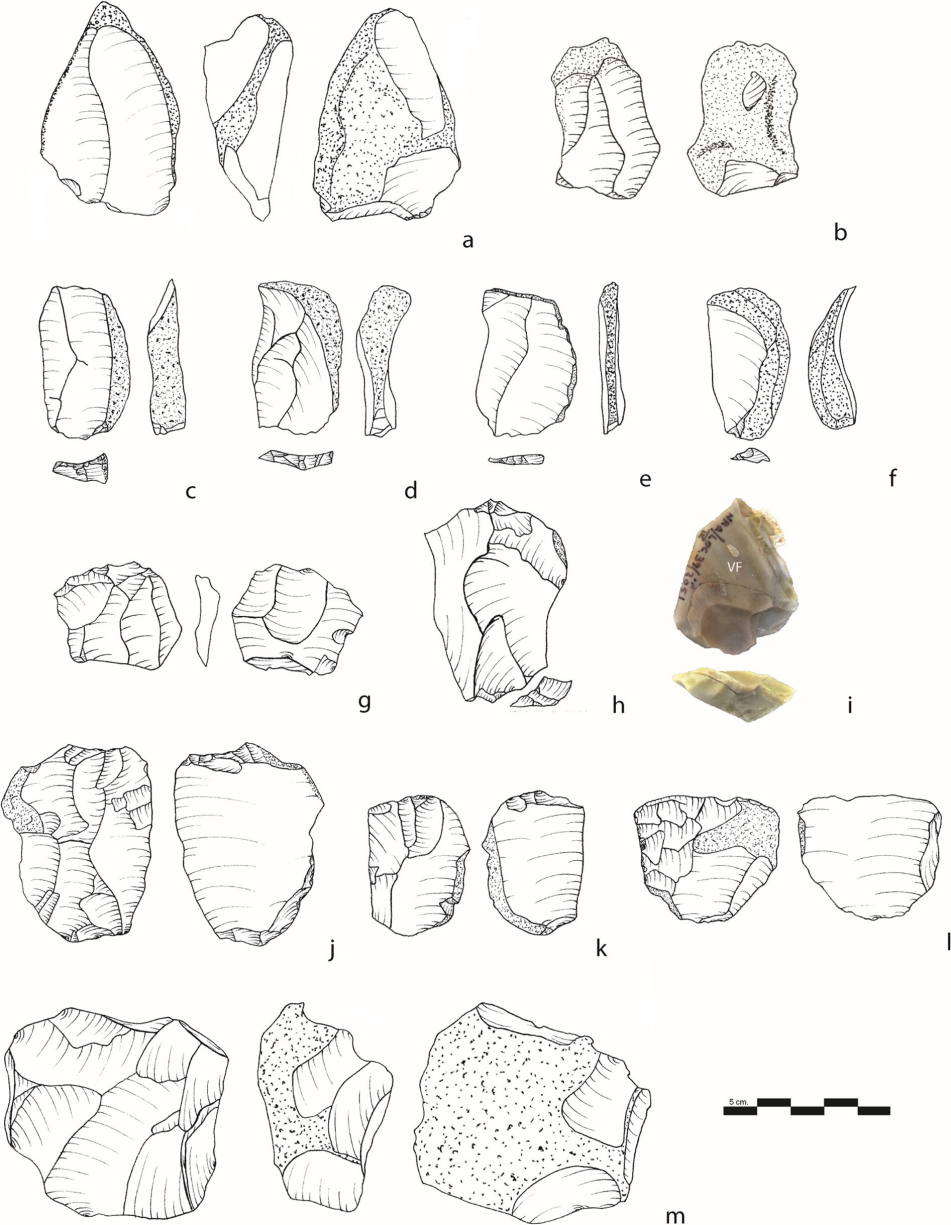
Cores. a, b: NBKC (Naturally Backed Knife Production Core); c, d, e, f: NBKs (Naturally Backed Knives); g, h: Core-on-flakes; i: Refits of a core-on-flake and Kombewa flake; j, k, l: Nahr Ibrahim pieces; m: A hierarchical surface core that possibly represents a preform of a Levallois core.

**Fig 9:**
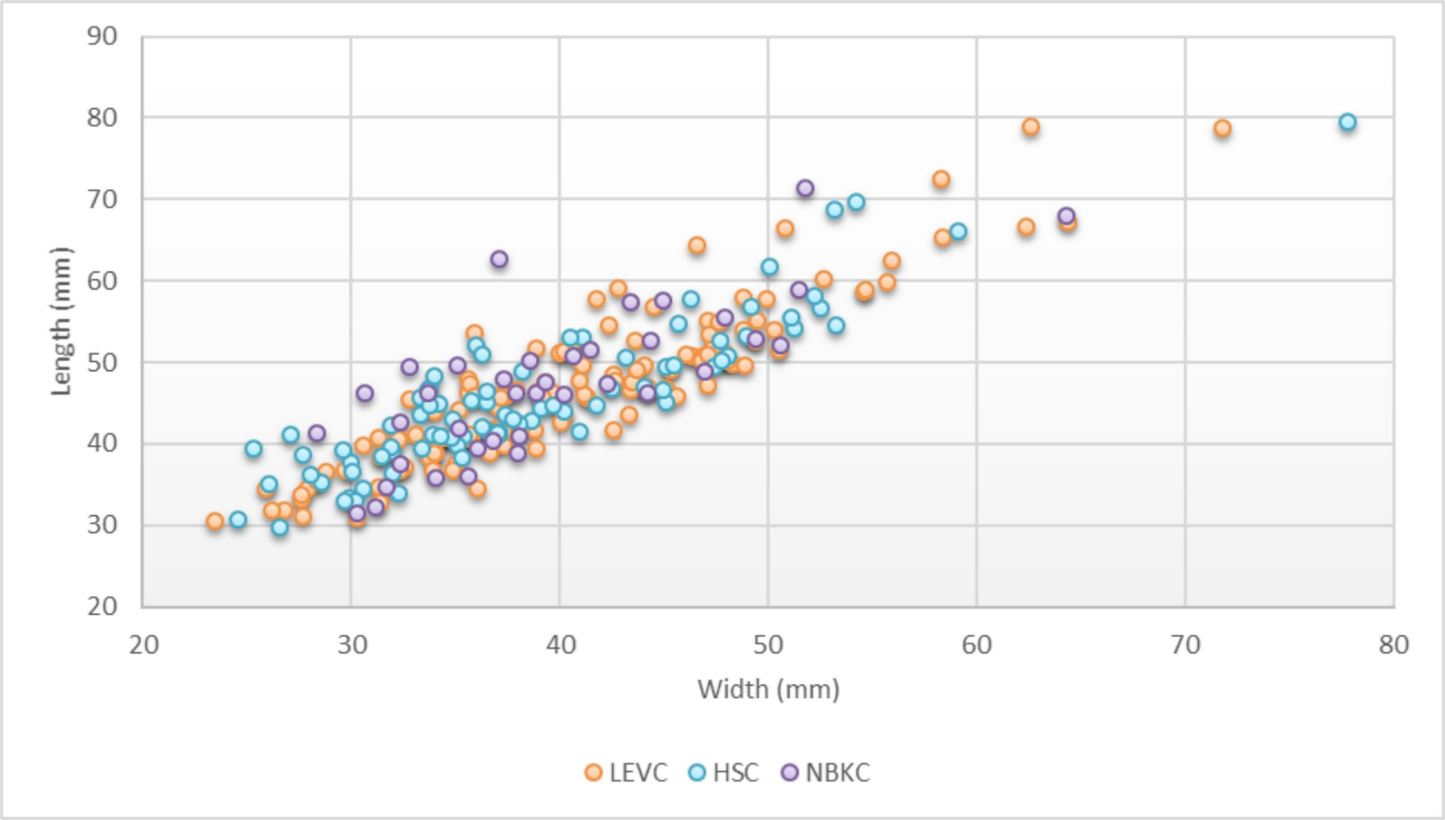
The length and width distribution of core types. LEVC = Levallois cores; NBKC = NBK production cores; HSC = Hierarchical surface cores.

A series of cores bearing comparable technical characteristics have been identified as a “NBK production core” (i.e., NBKC) (n = 39, 9.1% of the total core sample). The definition and interpretation are based on the observations made on cores and products during this study and in previous accounts at Nesher Ramla [47,50]. The NBKCs exhibit a Levallois-like volumetric conception with two hierarchical surfaces: one upper flaking surface and a striking platform [50] (Fig 8). The general morphology of these cores and the organization of their scar patterns may, at first look, be similar to the recurrent unidirectional parallel Levallois method, but the main difference lies in the desired end-product. The recurrent unidirectional Levallois methods aim at producing elongated Levallois flakes from the center of the debitage surface [74]. Such end-products are virtually absent in our assemblage. In contrast, in the NBKC reduction sequence described here, the goal was to exploit the lateral edges of the cores by the production of cortically backed pieces, while the central part of the débitage surface was not exploited. It is worth noting that a clear selection of small to medium-sized nodules has been observed for NBKCs. Their shape does not allow a long and multiple series of removals. They could not represent highly reduced Levallois cores or Levallois cores on initial stages of reduction, as indicating by their size distribution (Fig 9), which is similar to other hierarchized core types. Ninety percent of NBKCs exhibit between 2 and 4 scars with cortical lateral edges, indicating that the resulting products were NBKs. These products are widely represented within the débitage assemblage (9.3%; Fig 8).

The average length of the negative scar is the longest (38.9 mm) compared to the other types of cores (Table 2), revealing the desire to produce long rather than wide blanks. The striking surface is usually covered by cortex, and the flaking surfaces were exploited by unidirectional parallel and bidirectional methods (respectively 62% and 26%). Thus, most of the cores possesses a single striking platform (n = 25, 64%) and the others exhibit 2 striking platforms (usually opposed) (n = 14, 36%). The NBKCs exhibit a minimal investment in preparing the striking platform, the majority being plain or dihedral (n = 40; 75%).

#### The products

In general, NBKs exhibit a straight ventral profile associated with a facetted or plain striking platform (Table 4). All the NBKs possess a sub-triangular section and a straight or concave ventral profile (not twisted as observed on the NBKs originating from the unidirectional Levallois system [74]). There is an equal quantity of left and right NBKs, which in most cases exhibit unidirectional parallel negatives scars, followed by centripetal scar patterns (respectively, 58% and 18,2%; Table 4). The latter do not conform to the general unidirectional flaking pattern of the NBKCs and, may suggest that some NBKs represent core trimming elements of the Levallois system. This is also supported by the high number of NBKs per NBK cores (20/1), which is more than expected according to the number of scars on NBKC flaking surfaces.

### Other hierarchized reduction cores

The “Hierarchical Surface Core” category (24,5%) encompasses a large diversity of cores that possess the same characteristics: two non-changeable hierarchical surfaces, one acting as a striking platform and the other as a flaking surface. It shares some similarities with the Levallois system, such as the two distinct preparation and exploitation surfaces; however, the lack of convexity preparations, a lower investment in the striking platform preparation, and the non-preservation of a parallel plan of intersection are the main characteristics differing from the Levallois concept.

About a quarter of the cores with hierarchical surfaces could represent either previously exhausted (20%) or preforms of Levallois cores (4%). The cores identified as Levallois preforms are the biggest in the category (mean 61x56x38 mm) and exhibit a minimal preparation of the striking surface (Fig 8M). The cores identified as exhausted Levallois lost the criteria of the Levallois configuration due to the last-stage extra-flake removals. This type of knapping behavior was already described at Qafzeh [14].

The large majority of the sample (76%) represents simple hierarchized flake production. These cores, on average, are smaller than the Levallois cores (Table 2) but have larger ranges of dimensions (length 79.6–29.8 mm, width 77.8–24.6 mm, thickness 52.1–9.6 mm). These cores have a mean of 4.8 flake negatives on their flaking surface. The striking platforms are prepared (66% of facetted platform); however, a higher frequency of plain striking platforms is observed (19%) when compared to the Levallois core sample. Similar to the Levallois cores, the centripetal/orthogonal method is most frequently employed (53%), followed by the uni- and bidirectional flaking system (19% for both). The reduction pattern seems to be expedient with a low investment in preparation.

### Cores-on-flake

The Mishash flint dominates the assemblage of the core-on-flakes (COF; Fig 3). Even though several types of blanks were modified into COF, large non cortical and cortical flakes were preferred. The cortical elements appear to be longer and thicker than the other selected blanks. Different modalities of reduction are observed but, the unidirectional (37%) and centripetal (34%) methods prevailed. Ventral face (42%; Fig 8G and 8I), dorsal (32%; Fig 8H), or both (23%) surfaces were used for flaking. Facetted (63%), plain (20%), and dihedral (14%) are the most common types of striking platforms used and 3.77 flakes were removed in average. Differences appear when the types of COF raw material are considered; those made on “indeterminate” flint usually bear less cortex and exhibit more scar removals than the ones made on local Mishash flint (4.6 *vs*. 3.5).

#### The products

The Kombewa flakes result from flake removal from the ventral face of a COF. In the studied sample they are, on average, larger than the non-cortical flakes (Table 5). This suggests that Kombewa flakes were removed from large COFs, which are rarely found on site (Table 2). Kombewa flakes are characterized either by the presence of two ventral surfaces (52%) or two ventral surfaces associated with additional scars. Kombewa flakes are a common component of the Levantine MP assemblages; however, they generally appear in low frequency (between 1% and 4%) at Qafzeh, Amud, Quneitra, and Hummal [14,56,75,76]. In this regard, the Nesher Ramla assemblage follows the general trend (1.5% of the total assemblage).

### Nahr Ibrahim pieces

The Nahr Ibrahim (NI) technique was first described at the Nahr Ibrahim site in Lebanon [77]. In Unit III of Nesher Ramla, the NI technique is well-represented (0,7% of the total assemblage; 16% of the core assemblage) and is characterized by the removal of small flakes from a truncated-facetted striking platform created either on the distal (39%), proximal (35%), and less frequently on the lateral edge of the blank (Fig 8). The truncation was prepared on the ventral surface and the flakes were removed from the dorsal surface in 96% of the cases. Compared to the COFs, the Nahr Ibrahim secondary flake scars (mean of 3.5) are smaller and shorter. They often remove parts of the lateral edges of the flake (56%), in association with other removals located in the middle and, they less frequently appear solely in the middle of the blanks’ surface (34%).

### The retouched tools

The frequency of retouched tools in the lithic assemblage stands at 10.2%, which is high compared to other Levantine MP sites [14,78,79]. The retouched tool assemblage is dominated by scrapers (Table 6). The single side scraper is the most common tool type (45.9%); double scrapers, convergent scrapers, déjeté scrapers, and Mousterian points together represent 7.8%. The “retouched flake” category constitutes almost 18% of the assemblage and is characterized by either an irregular retouch or a retouch covering only a small part of the edge.

**Table 6:** Typological list of Nesher Ramla Unit III assemblage.

	TYPOLOGY (excluding the 2nd edge of composite tools)		With a lateral tranchet blow	
	n	%	n	%
Single convex side scraper	354	28.9%	140	40.1%
Single straight side scraper	104	8.5%	48	13.8%
Single concave side scraper	55	4.5%	20	5.7%
Single convex concave side scraper	16	1.3%	6	1.7%
Alternate retouch side scraper	7	0.6%		
Side scraper on ventral face	4	0.3%	1	0.3%
Side scraper with bifacial retouch	2	0.2%	1	0.3%
Double convex concave side scraper	8	0.7%	7	2.0%
Double convex side scraper	32	2.6%	21	6.0%
Double straight convex side scraper	8	0.7%	6	1.7%
Double straight side scraper	2	0.2%	2	0.6%
Transverse convex scraper	17	1.4%	4	1.1%
Transverse straight scraper	2	0.2%		
Transverse concave scraper	1	0.1%		
Convergent convex concave scraper	1	0.1%		
Convergent convex scraper	17	1.4%	7	2.0%
Convergent straight convex scraper	2	0.2%	1	0.3%
Déjeté scraper	13	1.1%	2	0.6%
Mousterian point	12	1.0%	2	0.6%
Retouched Levallois point	20	1.6%	5	1.4%
Raclette	58	4.7%	4	1.1%
Retouched on ventral face	7	0.6%		
Retouched flake	204	16.6%	28	8.0%
Retouched core	13	1.1%		
Notch	29	2.4%		
Denticulate	9	0.7%	1	0.3%
Endscraper	2	0.2%		
Beack	5	0.4%		
Truncation	5	0.4%		
Burin	5	0.4%		
Blanks with LTB	81	6.6%		
Indeterminate tool with LTB	42	3.4%	42	12.0%
Miscellaneous	6	0.5%		
Broken tool	83	6.8%	1	0.3%
TOTAL	1226	100%	349	100%

Upper Paleolithic (UP) tool types are nearly absent from the assemblage. The denticulate and notch categories constitute only 3.1% and multiple tools contribute to 7% of the retouched tools assemblage. We considered the most common retouch type in the assemblage as the main tool when classifying multiple tools (for instance, if a tool has a scraper retouch on one edge and a denticulate on the other, it will be classified as a scraper; following [14]). Single scrapers, retouched flakes, *raclettes*, and tools with a lateral tranchet blow are the most frequent combinations among multiple tools.

**Fig 10:**
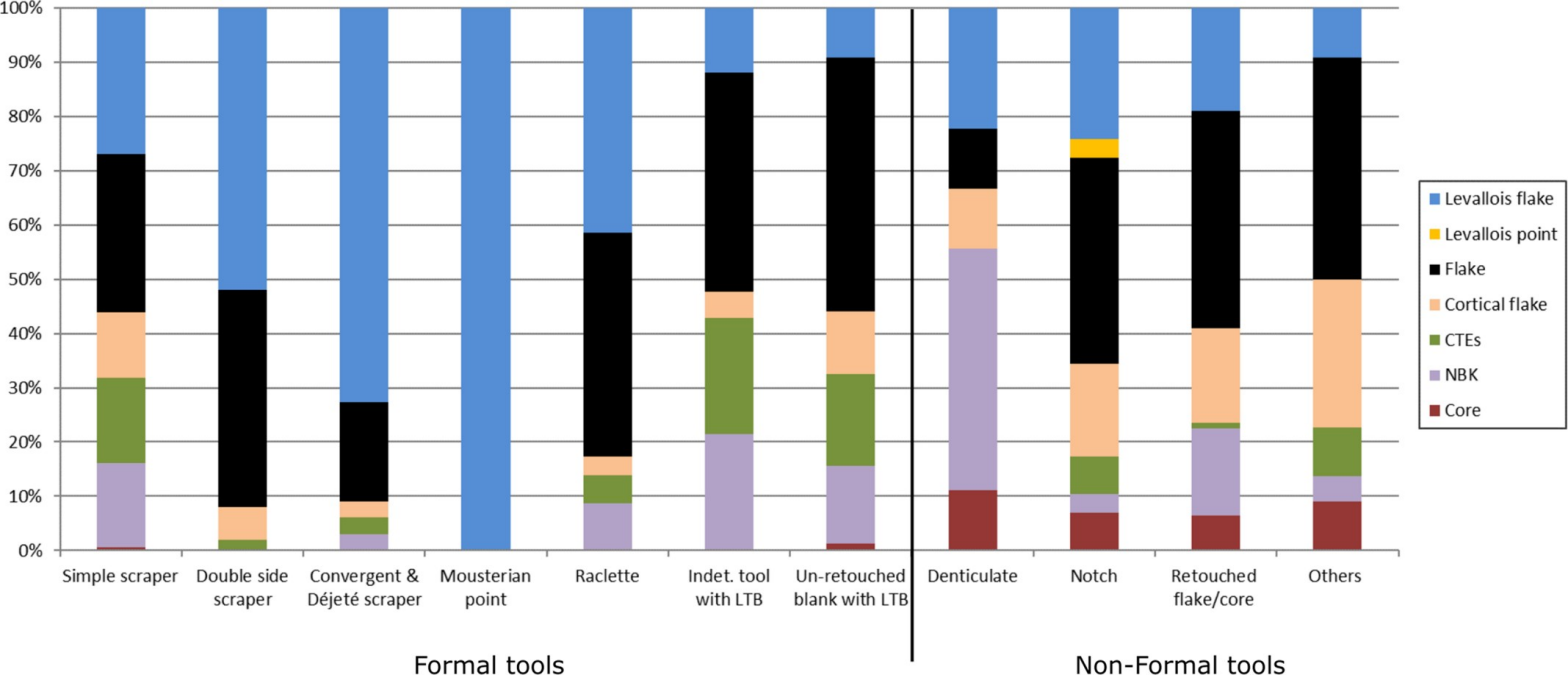
Typology according to the blank types. Correlation between typology and selected types of blanks.

**Fig 11:**
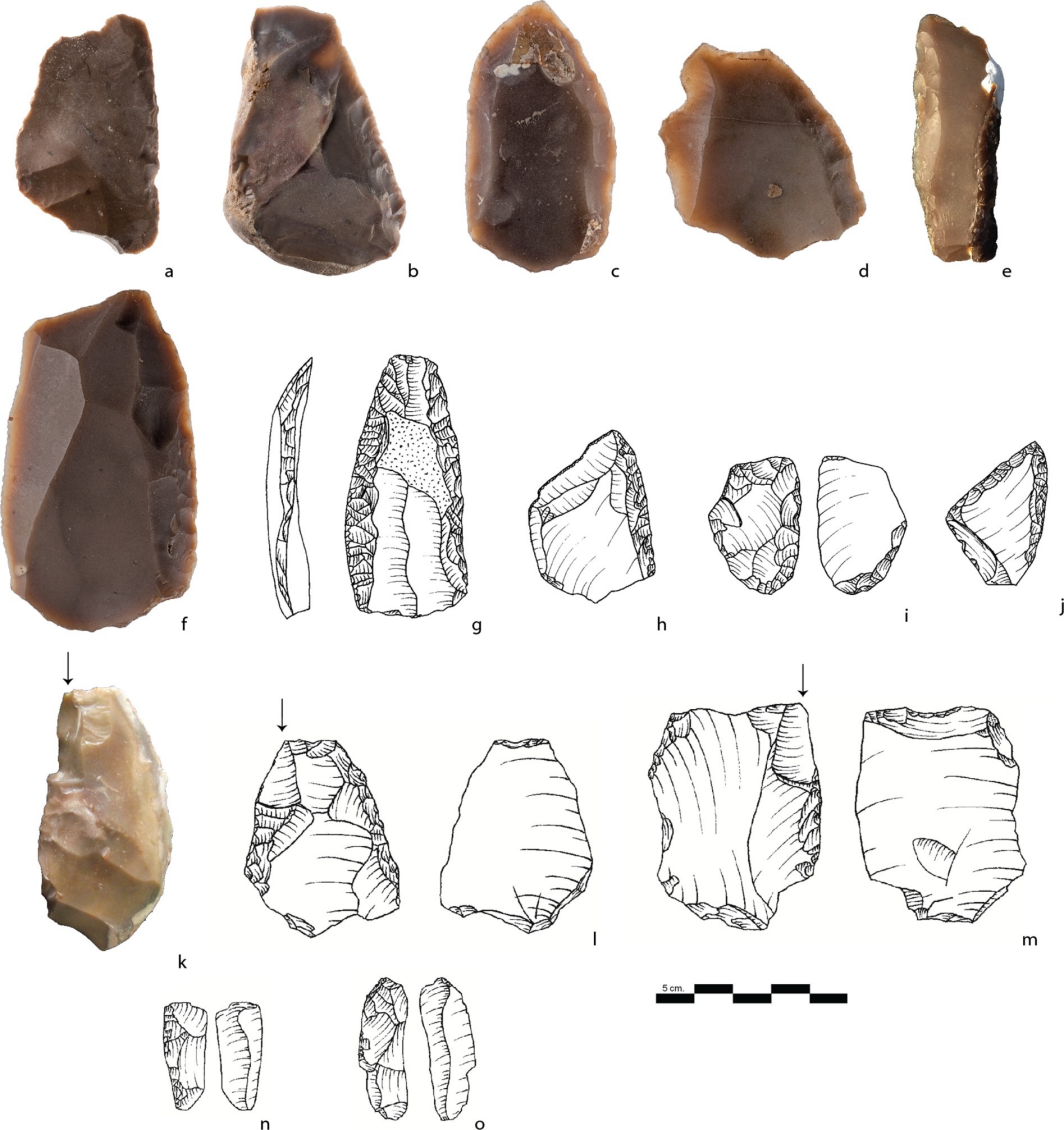
Retouched tools and spalls. a, b, c, d, e, f, h: simple side scrapers; g: convergent scraper. i: simple side scraper with truncation; j: déjeté scraper; k, l, m: scrapers with LTB; n-o: retouched LTB spalls. (Pictures a, b, c, d, f by T. Rogovski).

The non-cortical flakes and Levallois flakes were the most frequent blanks selected for retouch (32.2% and 23.8%, respectively), followed by the NBKs and the *débordant* flakes (Figs 10 and 11). The Levallois flakes are significantly more frequent among blanks for producing formal tool types (χ^2^ (4) = 19.028, *p* = .001). The non-formal tools were produced on a wider diversity of blanks (Fig 10). Similar to Qafzeh and Quneitra, UP tool types (end-scrapers and burins) are usually made on cortical flakes [14,56]. As a trend, already observed in many Levantine and European MP sites, the largest blanks were selected to be retouched [14] and references therein). Within the same blank category, all the retouched blanks are always longer than the unretouched ones (Table 5. Mann-Whitney test or the Levallois flakes *U* = 35862; z = 10.27; *p*< 0.05, for the flakes *U* = 52088; z = 16.038; *p*<0.05, for the NBK flakes *U* = 20100; z = 5.3369; p<0.05).

The tool assemblage is characterized by high retouch intensity (Fig 11 and S3 Table). The retouch is invasive and regular, standing out from the majority of Levantine MP sites that are usually characterized by unstandardized and non-invasive retouch [14]. The semi-Quina retouch is observed on 5% of the tools. Left and right edges were equally retouched (37.2% and 37.6%). Retouch is direct (92%) and rarely occurs on both edges (13.3%), or on the distal edge (6.6%) of the tools. Bifacial retouch is virtually absent from the tool sample. The convergent and déjeté scarpers possess, on average, the longest retouched edges and the more invasive retouch (S3 Table).

**Fig 12:**
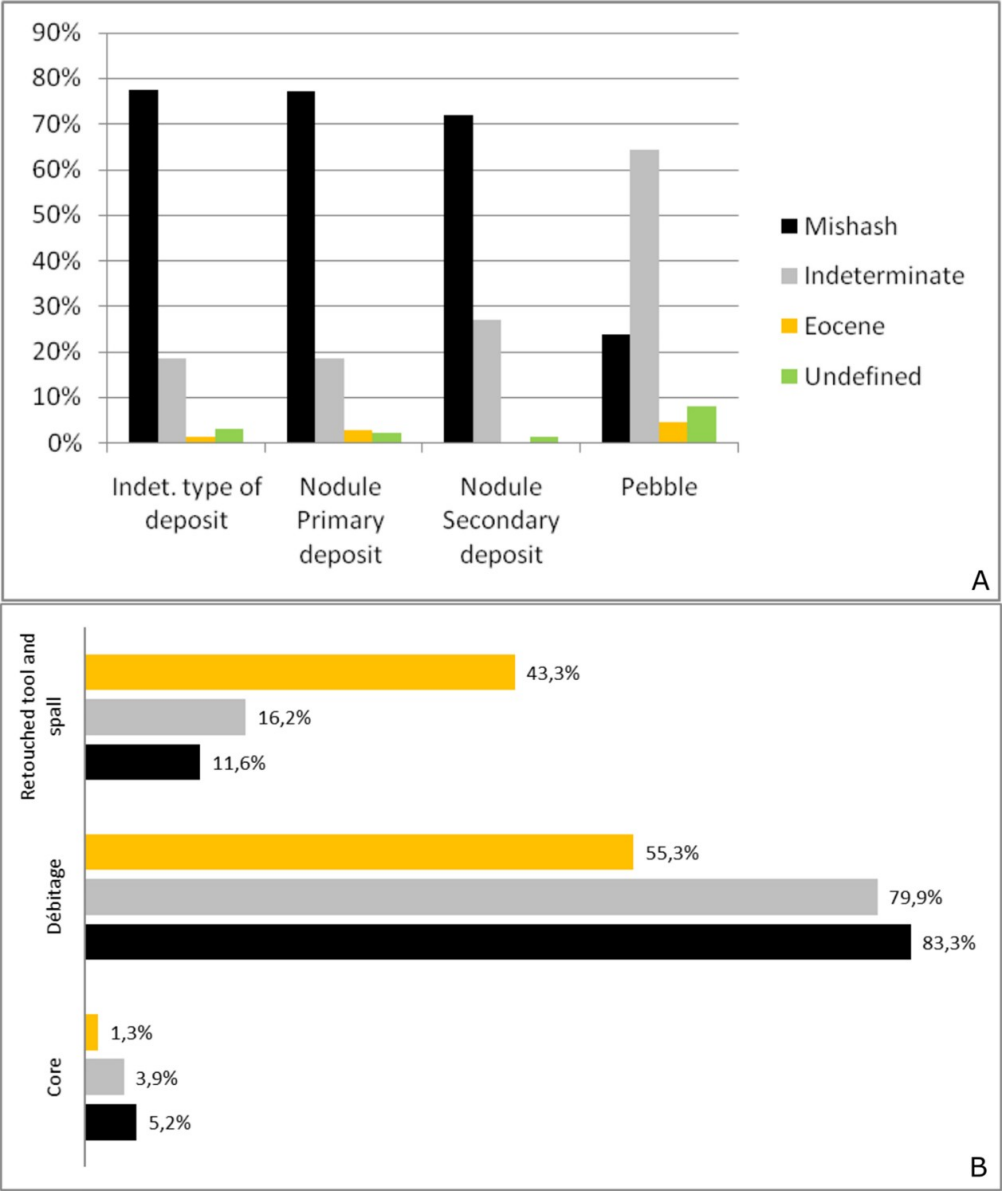
Flint raw materials characteristics. A: The frequency of flint types according to the morphology of the “nodules”; B: The frequency of flint types by major categories.

Finally, differences in raw material exploitation are clearly recognized within the retouched tool assemblage. Generally, the local Mishash flint dominates (63%) among the tools (Fig 3A–3E). However, the “indeterminate” and Eocene flint types are far better represented among the tools than other categories in the assemblage (Fig 12B). Furthermore, the most intensively retouched tools (i.e., the double scraper, convergent scraper, and Mousterian points) are made of Eocene and “indeterminate” flint (Fig 5E). In addition, all the scraper types made of “indeterminate” and Eocene flint are longer than the ones made of local Mishash flint (S4 Table).

#### Tools with a lateral tranchet blow

The retouch tool assemblage is characterized by the presence of numerous retouched tools with a lateral tranchet blow (LTB) on the retouch edges [80] (Fig 11). The assemblage includes both the tools and spalls resulting from the LTB. In addition, 1% of unretouched blanks (mostly flakes, CTEs, NBKs, and cortical flakes, respectively, 41%, 16%, 14%, and 11%) exhibit a scar of LTB.

The removal of a lateral tranchet spall follows a series of technical steps. First, a facetted truncation is created at the distal or proximal end of the blank (rarely on the lateral edge). The truncation served as a striking platform for removing the lateral tranchet spall.

In Unit III, the LTB technique appears mostly on scraper-like retouched edges (Table 6). Sometimes the LTB removed almost the entire retouched edge and, only a few retouch scars are still visible on the edge, making it impossible to identify the original tool type; therefore, these items were classified as “Indeterminate tool with LTB”. The LTB was more often transmitted from the distal end of the tool (60.2%), following a careful preparation of the truncation (90%). In 10% of the cases, the LTB was removed from a break or from a plain surface. Double LTB (either on the same edges or on opposite edges) appeared on 12% of the tools. Usually the “new” edge formed by the LTB remains raw and only in 2% of the cases it exhibits a new series of scraper-like retouch. A more common modification consists in the production of a short series of small retouches at the junction between the end of the LTB scar and the previously retouched edge (n = 140, 40%). They aimed at regularizing and flattening the hinge that developed at the distal end of the LTB scar [81]. In addition, small unidirectional flakes struck from the same striking platform and associated with the LTB were observed on 38% of the pieces. These removals aimed at flattening the convex dorsal surface and served as a possible guide for the LTB [82,83]. The new edge formed by the LTB removal exhibits irregular scars possibly formed during his used in 42% of the cases.

Retouched and non-retouched LTB spalls are also present in the assemblage (respectively, n = 216 and n = 75). They are identified by their double ventral faces (the previous and the new one, located on the lower surface), their elongated shape, and their retouched edge (Fig 11).

## Discussion

### Raw material transportation and exploitation

The study of the raw material exploitation patterns revealed that Mishash and “indeterminate” flint were exploited similarly, whereas Eocene flint represents a shorter on-site reduction sequence and an import of personal gear to the site (Fig 12B). The proportion of débitage elements and the extent of cortical remains on the artifacts, are the main proxies used to identify the degree of on-site knapping and the raw material imported to the site. According to the results of Levallois knapping experiments [54], the early phase of decortication should produce around 13% of cortical elements (50–100% of cortex). The cortical flakes (50%-100% of cortical cover) of Mishash and “indeterminate” flint types represent 12% of the entire flint assemblage, implying that first decortication phases took place at the site. The Eocene cortical flakes are less frequent (7%), suggesting that the decortication phase partially took place outside the site.

All the technical pieces of the reduction sequences are represented in a similar proportion for both Mishash and “indeterminate” flint assemblages (Figs 3 and 12B), indicating on-site knapping and retooling. However, the “indeterminate” flint is less frequent in comparison to the Mishash flint (Fig 3A) and, its primary origin is unknown. Some "indeterminate" flint artifacts were produced from pebbles (Fig 12A), which could have been possibly collected from nearby secondary sources. The ongoing refitting studies of the “indeterminate” flint assemblage indicate that, apart from the import of complete nodules or pebbles to the site, large and thick cortical flakes were also introduced, indicating the presence of a complementary raw material transport strategy. Furthermore, the frequency of intensively retouched tools within the "indeterminate" flint assemblage is high in comparison to the Mishash flint assemblage. This indicates that, in addition to the on-site lithic reduction, the exploitation of the “indeterminate” flint is also characterized by the import of blanks and retouched tools. Thus, the exploitation of the "indeterminate" flint might represent a mixed strategy of *provisioning of places* and *provisioning of individuals* [84].

The Eocene flint type is frequent within the retouched tools categories, and especially among the intensively retouched tools, e.g. double side-scrapers, convergent scrapers and Mousterian points (Fig 3E). Furthermore, the retouch on the Eocene scrapers (all categories) is more invasive (7.6 mm vs. 6.5 mm for Mishash-type tools; S3 Table). In addition, Eocene cores are virtually absent from the assemblage and core management elements are extremely rare, suggesting sporadic core reduction at the site and a high proportion of introduced elements.

The extensively retouched tools and the Levallois flakes of Eocene flint may represent curated components related to a *provisioning of individual* strategy [84]. These implements were more mobile and moved from one place to another, compared to other blank types (for example, non-cortical flakes and cores). Their presence on site may indicate a possible loss or their replacement by a new “personal gear” [84].

### The Levallois reduction system at Nesher Ramla

**Fig 13:**
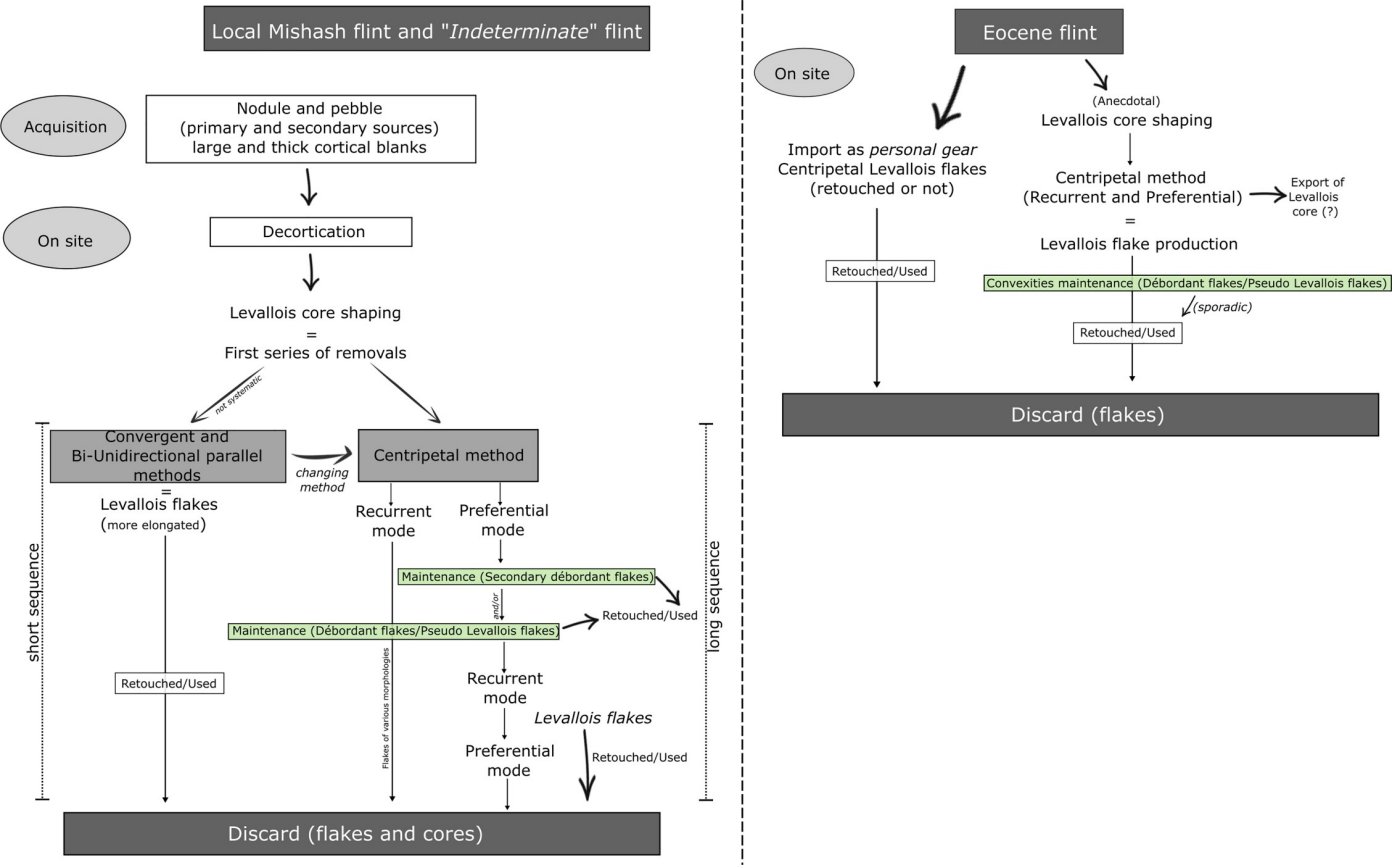
Levallois reduction sequences. Suggested schematic Levallois reduction sequences by raw material types observed in Unit III.

The Levallois centripetal flaking system dominates the flaking activity in Unit III. Fig 13 presents the Levallois reduction sequences by types of raw material, suggesting that a similar strategy with on-site knapping was used for the local Mishash and the “indeterminate” flint types and, that short reduction sequences were used for the Eocene flint.

Several aspects of the assemblages led us to hypothesize that shifting between preferential and recurrent modes (and *vice versa*) occurs through the knapping sequence. The recurrent system seems to have been commonly used throughout the Levallois core reduction (as seen from the full range of sizes of both cores and flakes), whereas the preferential mode seems to have been used more intensively at certain stages of the reduction sequence. At the beginning of the reduction sequence, when the core was still large enough to remove invasive, large, and symmetric Levallois flakes, the preferential mode may have been favored. This is manifested by the presence of “secondary” *débordant* flakes that are, on average, larger than the primary *débordant* flakes (Table 5), suggesting that they were struck at the beginning of the reduction. In addition, the sample yielded several small preferential Levallois cores, measuring between 3 and 4 cm, presenting a last preferential flake scar that removed almost the entire flaking surface. This knapping phenomenon is common during the MP and has been described in the Levant and Europe [14,85–88].

At Nesher Ramla, both recurrent and preferential modes of reduction were complementary and alternatingly used through the reduction system that aimed at maximizing the flake production (Fig 13). The knappers were able to switch between modes of production, depending on the state of the flaking surfaces and benefit from the existing suitable convexities to produce different types of products. Moreover, the analyses of the dorsal scar patterns on the Levallois flakes, combined with metrical observations and the extent of cortex suggest that, in some cases, flakes with unidirectional, bidirectional, and convergent scar patterns were removed at the beginning of the Levallois reduction sequence, while, on more advanced stages of knapping, the centripetal method was favored (Fig 13).

### The place of Unit III Nesher Ramla, within the Levantine Middle Paleolithic

The Unit III lithic assemblage shares similarities with other mid-MP assemblages dated to MIS 5. The Qafzeh Cave terrace (layers XXIV-XV) [14] was thoroughly analyzed and shows many similar characteristics to Unit III of Nesher Ramla. At both sites, the assemblages are characterized by; the use of both preferential and recurrent modes for producing wide rectangular/circular Levallois flakes, by the presence of characteristic Levallois by-products (e.g., primary and secondary *débordant* flakes, pseudo-Levallois points and flakes) and, by the low frequency of Levallois points (except for levels XV and VIIa in Qafzeh Cave) produced by the unidirectional convergent method. Qafzeh assemblage has also yielded NBKs, but in smaller frequencies than in Nesher Ramla [50]. The NBKs in Qafzeh were interpreted as core trimming elements, and mainly related to the recurrent unidirectional convergent Levallois method and, to a lesser extent to the recurrent centripetal method [14]. At Nesher Ramla, the presence of specific cores for NBK production, the large quantity of NBKs, their morphological and technological characteristics, as well as the absence of the Levallois unidirectional parallel exploitation system and elongated Levallois elements, suggest the presence of a specific reduction sequence aimed at the NBK production.

The general description of the assemblages and the drawings of lithic artifacts at Nahr Ibrahim, Naamé, and Ras El-Kelb indicate the prevalence of the Levallois centripetal flake production that was sometimes associated with the production of Levallois points [15,16,18,19]. However, in absence of detailed analyses and especially description of the by-products, detailed comparisons are difficult to make. Recent studies [20,21] enable better comparisons with the site of Skhul, which displays some similarities with Unit III. These include the prevalence of the centripetal method for Levallois flake production, the higher number of preferential Levallois cores compared to recurrent ones, the high frequency of core-on-flakes, the large number of retouched tools, and the low frequency of Levallois cores for points. On the other hand, Skhul assemblages exhibit a higher frequency of Levallois points and a lower frequency of core trimming elements and cortical pieces [21]. However, the results from Skhul are biased by the post-excavation artifact selection and should be used with caution [21].

Among the special features that distinguish between Unit III of Nesher Ramla and other Levantine MP sites, are the retouched tool component and the NBK production. Unlike the lithic assemblage of Nesher Ramla that is characterized by high frequency of retouched tools and by an abundance of intensively retouched tools, a generally observed pattern in the Levantine MP assemblages suggests a low frequency of retouched tools and a low intensity of retouch [14,78]. Furthermore, the use of the LTB technique is a unique characteristic of the Nesher Ramla retouched tool assemblage. This technique has been described in various techno-complexes of the Lower and Middle Paleolithic and, in various geographical areas [81,82,89–93], but was never systematically used in the Levantine MP. This specific technical process may reflect an innovative functional and/or cultural behavior.

Intra-site comparisons cannot be fully carried out since only a part of the entire lithic assemblage of Nesher Ramla have been studied in detail. From the preliminary data, the lithic technological organization does not reveal strong variations in the upper part of the sequence (units I-III) [50]. The Levallois centripetal method, the NBKC reduction strategy as well as the production of LTB were also identified in units I and II. Units IV-VI are still under study.

### The centripetal Levallois system: A geographical and temporal overview

The Levallois system, both recurrent and preferential modes, has occurred as early as 300 kya (the end of MIS 9, the beginning of MIS 8) in several locations in Western Europe and Africa and around 250 kya in the Levant [10,13,94–99]. The development of the Levallois system is usually considered as a hallmark of the Lower to Middle Paleolithic transition (and Early to Middle Stone Age “MSA” transition in Africa) and is seen as a behavioral change expressed by a shift from bifacial technology to a hierarchized pre-planned knapping system for flake/point and blade production [97,98,100]. Often, the Levallois system is associated with different types of assemblages (handaxes/bifacial shaping) and is usually accompanied by other reduction methods (i.e., Discoid, “*système par surface de débitage alternée*” (SSDA) and other expedient and core-on-flakes reductions) [101–106].

Formulating a general picture and a scope of centripetal recurrent Levallois at the end of MIS 6 and beginning of MIS 5 is challenging due to the different employed nomenclatures (e.g., prepared core, discoid, unifacial radial system, etc.), the different analytical approaches employed (e.g., lack of distinction between the preparation and exploitation stages), the emphasis made on the cores at the expense of products and by-products, and finally due to the composition of the assemblages themselves (e.g., the lack of some technological items).

**Fig 14:**
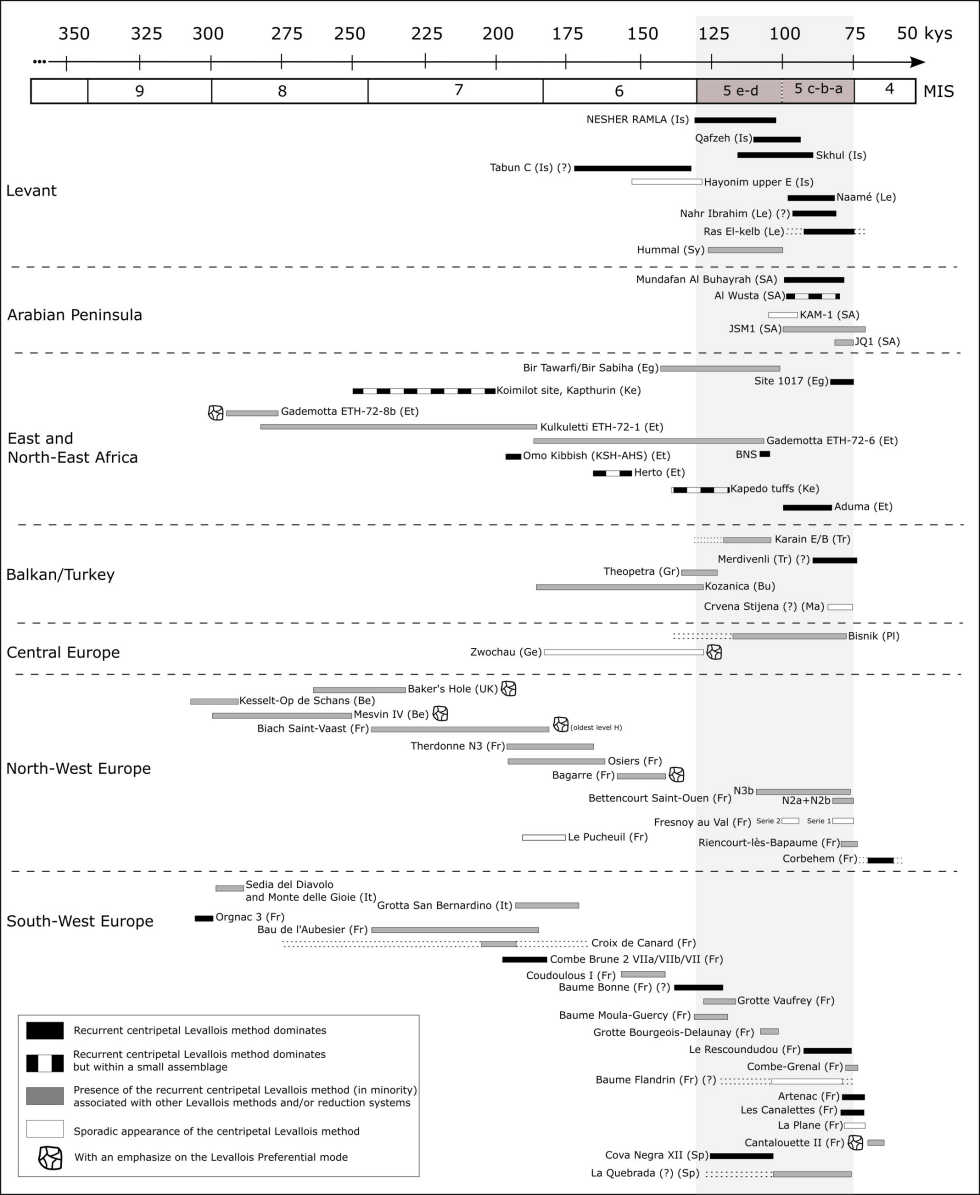
Geographical and temporal distribution of the Levallois centripetal system. Is = Israel, Le = Lebanon, Sy = Syria, SA = Saudi Arabia, Om = Oman, Eg = Egypt, Ke = Kenya, Et = Ethiopia, Tr = Turkey, Gr = Greece, Bu = Bulgaria, Ma = Macedonia, Pl = Poland, Ge = Germany, UK = United Kingdom, Be = Belgium, Fr = France, It = Italy, Sp = Spain. (S5 Table).

Fig 14 presents the Middle and Upper Pleistocene sites of Europe, Levant, Arabia, and Eastern Africa, which exhibit evidence for the use of the centripetal Levallois flaking system (S5 Table). During the early phases of MP in Europe and MSA in East Africa, the presence of the centripetal Levallois system was already observed, with some emphasis on the preferential mode [107,108], but almost never as the dominant reduction method. In Western Europe, the Levallois recurrent unidirectional parallel, unidirectional convergent and bidirectional methods for flake, blade and sometimes point production were more common during the EMP [105,106,109–112], where they occur together with other reduction sequences such as the discoid and other non-Levallois systems for flake and blade production [104,107,113]. Sites dated to the first half of MIS 5 (130–100 ka) are relatively few, and even though it was argued that the recurrent centripetal method became more frequent in the MIS 6 and 5 periods [88,107,114], the published data indicate that during this period the centripetal method was only sporadically applied and was never a dominant reduction sequence (Fig 14 and S5 Table). The East African record during the MIS 5 is scarce and the Levallois centripetal method often occurs with other methods of reduction [115,116].

On the contrary, the Levant and in a lesser extent the Arabian Peninsula, are the only regions where the centripetal Levallois method is a dominant reduction strategy during this time span [14,21,26,29,31,50,117,118]. At Nesher Ramla and in other Levantine MIS 5 sites, the lithic record shows evidence for a well-developed use of the Levallois centripetal system, including all the classical products of the core utilization and maintenance, which are rare in the East African and European record. It is important to note that the Levallois centripetal system was not part of the EMP lithic behavior in the Levant [8,10–12]. Thus, while dominant in the mid-MP techno-complex, the Levallois centripetal system does not show continuous regional development from the EMP and is likely to be originated elsewhere.

## Conclusions

The Nesher Ramla karst sinkhole contains one of the richest lithic assemblage dated to the end of MIS 6 and MIS 5. Although the present study covers only a single unit of the site, it significantly contributes to the understanding of the Levantine mid-MP techno-complex. The study suggests that complete reduction sequences took place at the site, from the initial decortications to the retouching and retooling activities. This strategy was supplemented by introduction of already finished tools made on non-local raw materials. The Levallois flaking system dominates the assemblage, and the desired flake morphotypes were mainly produced by the recurrent and preferential centripetal methods. Levallois points represent a minimal component of the toolkit. Intensively retouched scrapers and tools with LTB are dominant in the retouched tools assemblage of Nesher Ramla Unit III. The special technological traits of the assemblage that do not occur in other Levantine MP sites are the production of NBKs as end-products and the frequent use of the LTB.

A major technological characteristic, common to many contemporaneous sites from the Levant and neighboring regions, is the development and the extensive use of the Levallois centripetal method. This trend differs from what is usually observed in Europe and Africa, where the centripetal Levallois method is modestly represented during MIS 5 and always occurs along other more dominant knapping methods. This study provides additional evidence that MIS 5 sites in the Near-East possess common technological characteristics, especially the dominance of the centripetal Levallois method. Nonetheless, we demonstrate that inter-site variability occurred during this period as, for instance, expressed at Nesher Ramla by the LTB technical process and the NBK production. This variability can be explained by several parameters such as the site functions, the raw material constraints or, the presence of different populations.

## Supporting information

S1 Table Limestone assemblage. (DOCX)

S2 Table Manuport and percussion tools assemblage. (DOCX)

S3 Table Length and deepness of the retouch on different types of scrapers. (DOCX)

S4 Table Dimensions of tools according to the blank types and raw material. (DOCX)

S5 Table Chrono-temporal cluster of sites displaying the Levallois centripetal method. (DOCX)

S1 Fig Levallois systems according to different modes, methods, exploitation and preparation. (DOCX)

S2 Fig Levallois cores. Yellow color illustrates last additional flake removal. Grey color illustrates preparation flakes (predetermining).
(DOCX)

S3 Fig Schematic illustrations of pseudo-Levallois/points removals and débordant flake removal on a centripetal Levallois core flaking surface. 1. Pseudo-Levallois points/flakes can generate different scar patterns organization according to their place on the surface. 2. Position of a débordant flake and schematized scar pattern.
(DOCX)
