# Supplementary material for: New insights into early MIS 5 lithic technological behavior in the Levant: Nesher Ramla, Israel as a case study
Source: PLoS One. 2020 Apr 3;15(4):e0231109. doi: 10.1371/journal.pone.0231109 (PMC7122790; doi:10.1371/journal.pone.0231109)
Supplement: S1 Table — (DOCX) [file pone.0231109.s001.docx]

S1 Table. Limestone assemblage.

| Limestone Assemblage | N | % |
| --- | --- | --- |
| **Core** | 6 | 5% |
| **Flake** | 39 | 35% |
| **Chunk** | 30 | 27% |
| **Entame*** | 32 | 29% |
| **Tool** | 1 | 1% |
| **Chopper** | 2 | 2% |
|  | 110 | 100 |

*could derive from débitage or breakage during percussion
