# Supplementary material for: New insights into early MIS 5 lithic technological behavior in the Levant: Nesher Ramla, Israel as a case study
Source: PLoS One. 2020 Apr 3;15(4):e0231109. doi: 10.1371/journal.pone.0231109 (PMC7122790; doi:10.1371/journal.pone.0231109)
Supplement: S2 Table — (DOCX) [file pone.0231109.s002.docx]

S2 Table. Manuport and percussion tools assemblage.

| Manuport | | n | % |
| --- | --- | --- | --- |
| Percussion tools | Anvil | 15 | 5% |
|  | Hammerstone | 138 | 42% |
|  | Splitted pebble | 9 | 3% |
|  | Pebbles with striations and/or polish | 9 | 3% |
| Manuport (un-worked) | Limestone river pebble | 148 | 45% |
|  | Flint pebble | 6 | 2% |
|  | Indeterminate pebble | 1 | 0% |
| **Total** |  | 326 | 100 |

In addition to the knapped limestone assemblage, a large sample of manuports was retrieved from Unit III, weighing to more than 120 kg. River pebbles and blocks of limestone, and few examples of flint (5%), were brought to the site. Preliminary observations of the surfaces damages, show presence of different types of percussion marks and wear, which allow us to make a first classification of the worked materials. Hammerstones and anvils were identified in a large quantity as well as “unmodified” pebbles which could represent an economic/storage strategy or pieces which was lightly or shortly used. Limestone pebbles and blocks are abundant in the nearby Ayalon stream, however additional petrographic analyses are needed to confirm their provenience.
