# Supplementary material for: New insights into early MIS 5 lithic technological behavior in the Levant: Nesher Ramla, Israel as a case study
Source: PLoS One. 2020 Apr 3;15(4):e0231109. doi: 10.1371/journal.pone.0231109 (PMC7122790; doi:10.1371/journal.pone.0231109)
Supplement: S3 Table — (DOCX) [file pone.0231109.s003.docx]

S3 Table. Length and deepness of the retouch on different types of scrapers.

| Retouch edge length | Mean | SA | deepness retouch mm (mean) |
| --- | --- | --- | --- |
| Single side-scraper | 35,46 | 15,61 | 6,78 |
| Retouch flake | 23,98 | 15,62 | 3,45 |
| Raclette | 29,46 | 15,69 |  |
| Denticulate | 28,87 | 15,68 |  |
| Transverse scraper | 31,97 | 15,64 | 5,75 |
| Double scraper (edge1) | 32,26 | 16,21 |  |
| Double scraper (edge2) | 31,45 | 15,19 | 7,46 |
| Double scraper (both edges) | 31,85 | 15,74 |  |
| Convergent scraper (edge1) | 40,86 | 16,04 |  |
| Convergent scraper (edge2) | 45,86 | 15,45 |  |
| Convergent scraper (both edges) | 43,36 | 15,77 | 8,29 |
| Déjeté scraper | 43 | 15,86 |  |
| Single convex scraper | 36,2 | 15,61 |  |
| Single concave scraper | 31,3 | 15,64 |  |
| Single straight scraper | 32,06 | 15,61 |  |
| Single convex concave scraper | 41,54 | 15,56 |  |
| Single scraper Mishash | 34,13 | 15,61 |  |
| Single scraper Eocene | 31,33 | 15,09 |  |
| Single scraper indeterminate flint | 39,09 | 15,66 |  |
| Double scraper Mishash | 27,58 | 15,68 |  |
| Double scraper indeterminate flint | 34,66 | 15,74 |  |
| Double scraper Eocene | 35,11 | 15,58 |  |
