# Supplementary material for: New insights into early MIS 5 lithic technological behavior in the Levant: Nesher Ramla, Israel as a case study
Source: PLoS One. 2020 Apr 3;15(4):e0231109. doi: 10.1371/journal.pone.0231109 (PMC7122790; doi:10.1371/journal.pone.0231109)
Supplement: S4 Table — (DOCX) [file pone.0231109.s004.docx]

S4 Table. Dimensions of tools according to the blank types and raw material.

|  | Length mm | Width mm |
| --- | --- | --- |
|  | mean | mean |
| All single side scrapers | 52,43 | 35,49 |
| Mishash: single side scrapers | 51,6 | 34,93 |
| Indeterminate flint: single side scrapers | 54,33 | 36,5 |
| Eocene flint: single side scrapers | 52,4 | 36,1 |
| All double side scrapers | 52,96 | 36,53 |
| Mishash: double side scrapers | 47,75 | 36,05 |
| Indeterminate flint: double side scrapers | 54,78 | 35,7 |
| Eocene: double side scrapers | 60,31 | 41,28 |
| All convergent scrapers | 57,08 | 38,66 |
| Mishash: convergent scrapers | 52 | 37,7 |
| Indeterminate flint: convergent scrapers | 58,03 | 43,97 |
| Eocene: convergent scrapers | 59,24 | 37,15 |
| All déjeté scrapers | 47,3 | 46,31 |
| Mishash: déjeté scrapers | 39,06 | 45 |
| Indet and Eocene flint: déjeté scrapers | 55,54 | 47,62 |
