# Supplementary material for: New insights into early MIS 5 lithic technological behavior in the Levant: Nesher Ramla, Israel as a case study
Source: PLoS One. 2020 Apr 3;15(4):e0231109. doi: 10.1371/journal.pone.0231109 (PMC7122790; doi:10.1371/journal.pone.0231109)
Supplement: S5 Table — (DOCX) [file pone.0231109.s005.docx]

S5 Table. Chrono-temporal cluster of sites displaying the Levallois centripetal method.

| **REGION** | **SITE** | **COUNTRY** | **UNIT/LEVEL** | **MIS** | **DATING** | **REFERENCES** |
| --- | --- | --- | --- | --- | --- | --- |
| Levant | Nesher Ramla | Israel | Unit I-VI | end MIS 6-MIS 5 | 160±8-78±6 kyr (OSL) | [1–3] |
|  | Qafzeh | Israel |  | MIS 5 | 92 ± 5 kyr (TL)  115 ± 15 kyr (ESR) | [4–6] |
|  | Shkul | Israel | level B | MIS 5 | 119±18 (TL), 81±15-101±12 kyr(ESR) | [7,8] |
|  | Naamé | Lebanon |  | end MIS 5-MIS 4 | 92 kyr | [9] |
|  | Tabun | Israel | layer C (Unit I) | end MIS 6-MIS 5 | 134±8-170±10 (TL) 112±29-143±37 kyr (ESR) | [7,10,11] |
|  | Hummal | Syria | HM-B (5e, 5f1, 5f2, 5g) | MIS 5 | 128-98 kyr | [12] |
|  | Nahr Ibrahim | Lebanon | Central gallery | end MIS 5-MIS 4 | 80 or 92 kyr | [13–17] |
|  | Hayonim | Israel | Upper E level | end MIS 6 | 156-135 kyr | [18] |
|  | Ras El-kelb | Lebanon |  | end MIS 5-MIS 4 |  | [19] |
| Arabian Peninsula | Jebel Umm Sanman (JSM1) | Saudi Arabia |  | MIS5c-MIS4 | 100-60 kyr | [20,21] |
|  | Jebel Qattar (JQ1) | Saudi Arabia | unit B | MIS 5a |  | [20,22] |
|  | Mundafan Al Buhayrah | Saudi Arabia |  | Late MIS 5 | 100-80 kyr | [22,23] |
|  | Al Wusta | Saudi Arabia |  | Late MIS 5 | 95-86 kyr | [24] |
|  | KAM sites (1+4) | Saudi Arabia | surface | MIS 5 | 117±8-99±7 kyr | [25,26] |
| North and East Africa | Bir Tarfawi/Bir Sahara | Egypt |  | end MIS 6-MIS 5 | 140-100 kyr ? | [22] |
|  | Site 1017 | Egypt |  | end MIS 5 |  | [27] |
|  | Kapthurin formation | Kenya | Koimilot site | MIS 7 | 250-200 kyr | [28] |
|  | Gademotta and Hulkuletti | Ethiopia | ETH-721 | MIS 8-7-6 | 280-100 kyr | [29] |
|  | Omo Kibbish/ KSH | Ethiopia | KSH | MIS 7 | 195±5 kyr | [30] |
|  | Omo Kibbish/ AHS | Ethiopia | AHS | MIS 7 | 195±5 kyr | [30] |
|  | Omo Kibbish/BNS | Ethiopia | BNS | MIS 5 | 104±1 kyr | [30] |
|  | Herto | Ethiopia | Surface+excavation | MIS 6 | 160-154 kyr | [31] |
|  | Kapedo tuffs sites | Kenya | south localities | end MIS 6-MIS 5 | 135-123 kyr | [32] |
|  | Aduma | Ethiopia |  | MIS 5 | ~90 kyr | [33] |
| Turkey/Balkan | Karain E | Turkey | layers I6/I7 (complexe I/H) | MIS 5? | 120-110 kyr | [34–36] |
|  | Karain B | Turkey | PIV/PV/PVI | MIS 5? | 120 kyr | [36] |
|  | Merdivenli cave | Turkey | Layer I-V | End MIS 5 (?) |  | [37] |
|  | Theopetra cave | Greece | Layer II2-4 | end MIS 6-MIS 5 | 129±3-124±16 kyr | [38–40] |
|  | Kozarnika cave | Bulgaria | layers 10b; 9a-c (level XIV-XIII) | MIS 6 | 183±14-128±13 (OSL) 200-130 kyr (biochrono) | [41,42] |
|  | Crvena Stijena | Macedonia | levels XVII/XVIII | end MIS 5 |  | [43] |
| North and West Europe | Bisnik cave | Poland | layers 11-12-13-14 (A1/A2/A3) | end MIS 6-MIS 5 | 139±33-103 to 81±17 kyr (TL) | [44] |
|  | Markkleeberg | Germany | FCI | MIS 6/7/8 |  | [45,46] |
|  | Zwochau | Germany |  | MIS 6 |  | [45,46] |
|  | Kesselt Opde Schans | Belgium | ODS1/ODS2 | transition MIS 9/8 |  | [47,48] |
|  | Mesvin IV | Belgium |  | MIS 8 | 300-250 kyr | [49] |
|  | Baker's Hole | UK |  | end MIS 8-MIS 7 |  | [50,51] |
|  | Fresnoy au Val | France | Serie 2 | MIS 5c | 106,8±7,5 kyr | [52] |
|  |  |  | Serie 1 | MIS 5a |  |  |
|  | Bettencourt Saint Ouen | France | N3b | MIS 5d+a |  | [53,54] |
|  |  |  | N2a | MIS 5a |  |  |
|  |  |  | N2b | MIS 5a |  |  |
|  | Therdonne | France | N3 | end MIS 7 | 190-170 kyr | [52,55] |
|  | Biach Saint Vaast | France | H/IIA/II base/E/D20/D1/D | MIS 7 | 263±53-139±27 kyr | [56] |
|  | Osiers à Bapaume | France | serie B | MIS 7a-MIS 6 |  | [57] |
|  | Le Pucheuil | France | série B | MIS 6 | 200-180 kyr | [58] |
|  | Bagarre | France | couche 7 | MIS 6 |  | [59] |
|  | Corbehem | France | S1+terassement | MIS 4 |  | [59,60] |
|  | Riencourt les Bapaumes | France | level C.A. | end MIS 5 (?) |  | [61] |
| South and West Europe | Orgnac 3 | France | level 2 | MIS 8 | 302,9±2,9 kyr | [62,63] |
|  | Baume Bonne | France | ensemble IV | end MIS 6-MIS 5? |  | [64] |
|  | Baume Flandrin | France |  | MIS 5? |  | [62,65] |
|  | Baume Moula-Guercy | France | layer XV | MIS 5e | 120-130 krs | [66,67] |
|  | Bau de l'Aubesier | France | layers J-J3-J4 | MIS 7 |  | [68] |
|  | Combe Brune 2 | France | VIIa | end MIS 7- MIS 6 | 195-185 kyr | [63,69,70] |
|  |  |  | VIIb |  | 195-185 kyr |  |
|  |  |  | V-II |  | 195-185 kyr |  |
|  |  |  | VI |  | 195-185 kyr |  |
|  |  |  | X |  | 195-185 kyr |  |
|  | Croix de Canard | France | Level 3 | MIS 8 to 6 (?) | 190 kyr (?) | [63,69,71] |
|  | Grotte Vaufrey | France | layers VIII-IV | MIS 5e | 120±10 kyr | [72,73] |
|  | Le Rescoundudou | France |  | MIS 5e/c |  | [73–75] |
|  | Coudoulous I | France | layer 4 | MIS 6 | 200-126 kyr | [75,76] |
|  | Artenac | France | layers 8 to 5 (ens. 5) | end MIS 5 |  | [77] |
|  | Grotte Bourgeois-Delaunay (la chaise Vouthon) | France | level 10 | MIS 5 | 113±17 kyr (U/Th) | [78,79] |
|  | Les Canalettes | France | level 2 | MIS 4 | mean 73 kyr (TL) | [80] |
|  | Combe Grenal | France | level 36 | MIS 5a |  | [79,81] |
|  | Combe Grenal | France | level 6-7 | MIS 4/3 |  | [81] |
|  | Cantalouettes II | France | "la doline" | MIS 4 | 60 kyr | [82] |
|  | La Plane | France |  | end MIS 5- MIS 4 |  | [83,84] |
|  | Grotta Maggiore di San Bernardino | Italy | level VIII | end MIS 7-MIS 6 |  | [85] |
|  |  |  | level VII |  |  |  |
|  | Sedia del Diavolo and Monte delle Gioie | Italy |  | MIS 9-8 transition | 290-295 kyr | [86] |
|  | Cova Negra | Spain | XII-XI | MIS 5d/c | 117±17 kyr for XII, up to 255±20/206±23 kyr for XI | [87] |
|  | La Quebrada | Spain | VIII (lowest level) | MIS 5? |  | [88,89] |

**References**

1. Zaidner Y, Frumkin A, Porat N, Tsatskin A, Yeshurun R, Weissbrod L. A series of Mousterian occupations in a new type of site: The Nesher Ramla karst depression, Israel. Journal of human evolution. 2014;66:1–17.

2. Zaidner Y, Centi L, Prevost M, Shemer M, Varoner O. An Open-Air Site at Nesher Ramla, Israel, and New Insights into Levantine Middle Paleolithic Technology and Site Use. In: Nishiaki Y, Akazawa T, editors. The Middle and Upper Paleolithic Archeology of the Levant and Beyond [Internet]. Singapore: Springer Singapore; 2018. p. 11–33. Available from: https://doi.org/10.1007/978-981-10-6826-3_2

3. Guérin G, Valladas H, Joron J-L, Mercier N, Reyss J-L, Zaidner Y. Apports de la datation par la luminescence des sites du Proche-Orient et résultats préliminaires du site de Nesher Ramla (Israël). L’Anthropologie. 2017 May 18;121(1–2):35–45.

4. Schwarcz HP, Grün R, Vandermeersch B, Bar-Yosef O, Valladas H, Tchernov E. ESR dates for the hominid burial site of Qafzeh in Israel. Journal of Human Evolution. 1988;17(8):733–737.

5. Valladas H, Reyss J-L, Joron J-L, Valladas G, Bar-Yosef O, Vandermeersch B. Thermoluminescence dating of Mousterian Proto-Cro-Magnon’remains from Israel and the origin of modern man. Nature. 1988;331(6157):614–616.

6. Hovers E. The lithic assemblages of Qafzeh Cave. Oxford University Press; 2009.

7. Garrod DA, Bate DMA. The Stone Age of Mount Carmel. Volume 1: Excavations at the Wady El-Mughara. Clarendon Press, Oxford; 1937.

8. Mercier N, Valladas H, Bar-Yosef O, Vandermeersch B, Stringer C, Joron J-L. Thermoluminescence date for the Mousterian burial site of Es-Skhul, Mt. Carmel. Journal of Archaeological Science. 1993;20(2):169–174.

9. Fleisch H. Les habitats du Paléolithique Moyen à Naamé (Liban). Bulletin de la Musée de Beyrouth. 1970;23:25–93.

10. Grün R, Stringer C. Tabun revisited: revised ESR chronology and new ESR and U-series analyses of dental material from Tabun C1. J Hum Evol. 2000 Dec;39(6):601–12.

11. Mercier N, Valladas H. Reassessment of TL age estimates of burnt flints from the Paleolithic site of Tabun Cave, Israel. Journal of Human Evolution. 2003;45(5):401–409.

12. Hauck TC. Mousterian technology and settlement dynamics in the site of Hummal (Syria). J Hum Evol. 2011 Nov;61(5):519–37.

13. Solecki R. The Middle Paleolithic site of Nahr Ibrahim (Asfourieh Cave) in Lebanon. Problems in Prehistory: North Africa and the Levant, Southern Methodist University Press, Dallas, TX. 1975;283–295.

14. Porat N, Schwarcz HP. Use of signal subtraction methods in ESR dating of burned flint. International Journal of Radiation Applications and Instrumentation Part D Nuclear Tracks and Radiation Measurements. 1991 Jan 1;18(1):203–12.

15. Dockall JE. Wear Traces and Projectile Impact: A Review of the Experimental and Archaeological Evidence. Journal of Field Archaeology. 1997 Jan 1;24(3):321–31.

16. Dockall JE. Technological and functional variability of convergent tools from Nahr Ibrahim, Lebanon: behavioral implications for Levantine Mousterian technological organization [Internet] [PhD Thesis]. Texas A&M University; 1997 [cited 2019 Aug 23]. Available from: https://oaktrust.library.tamu.edu/handle/1969.1/158169

17. Monigal K. The Levantine Leptolithic: blade production from the Lower Paleolithic to the dawn of the Upper Paleolithic [PhD Thesis]. Southern Methodist University; 2002.

18. Meignen L. A preliminary report on Hayonim cave lithic assemblages in the context of the Near Eastern Middle Palaeolithic. Neandertals and Modern Humans in Asia. 1998;165–180.

19. Copeland L. The Middle Paleolitic of Adlun and Ras el Kelb (Lebanon): First Results from a Study of the Flint Industries. Paléorient. 1978;33–57.

20. Petraglia MD, Alsharekh A, Breeze P, Clarkson C, Crassard R, Drake NA, et al. Hominin Dispersal into the Nefud Desert and Middle Palaeolithic Settlement along the Jubbah Palaeolake, Northern Arabia. PLOS ONE. 2012 Nov 19;7(11):e49840.

21. Groucutt HS, Shipton C, Alsharekh A, Jennings R, Scerri EML, Petraglia MD. Late Pleistocene lakeshore settlement in northern Arabia: Middle Palaeolithic technology from Jebel Katefeh, Jubbah. Quaternary International. 2014;382:215–36.

22. Groucutt HS, Scerri EM, Lewis L, Clark-Balzan L, Blinkhorn J, Jennings RP, et al. Stone tool assemblages and models for the dispersal of Homo sapiens out of Africa. Quaternary International [Internet]. 2015 [cited 2015 Apr 24]; Available from: http://www.sciencedirect.com/science/article/pii/S1040618215000622

23. Crassard R, Petraglia MD, Drake NA, Breeze P, Gratuze B, Alsharekh A, et al. Middle Palaeolithic and Neolithic Occupations around Mundafan Palaeolake, Saudi Arabia: Implications for Climate Change and Human Dispersals. PLOS ONE. 2013 juil;8(7):e69665.

24. Groucutt HS, Grün R, Zalmout IAS, Drake NA, Armitage SJ, Candy I, et al. Homo sapiens in Arabia by 85,000 years ago. Nature Ecology & Evolution. 2018 May;2(5):800.

25. Rosenberg TM, Preusser F, Risberg J, Plikk A, Kadi KA, Matter A, et al. Middle and Late Pleistocene humid periods recorded in palaeolake deposits of the Nafud desert, Saudi Arabia. Quaternary Science Reviews. 2013 Jun 15;70:109–23.

26. Scerri EML, Breeze PS, Parton A, Groucutt HS, White TS, Stimpson C, et al. Middle to Late Pleistocene human habitation in the western Nefud Desert, Saudi Arabia. Quaternary International. 2014;382:200–14.

27. Goder-Goldberger M. The Khormusan: Evidence for an MSA East African industry in Nubia. Quaternary International. 2012;300:182–94.

28. Tryon CA. “Early” Middle Stone Age Lithic Technology of the Kapthurin Formation (Kenya). Current Anthropology. 2006 Apr 1;47(2):367–75.

29. Douze K, Delagnes A. The pattern of emergence of a Middle Stone Age tradition at Gademotta and Kulkuletti (Ethiopia) through convergent tool and point technologies. Journal of Human Evolution. 2015;91:93–121.

30. Shea JJ. The Middle Stone Age archaeology of the Lower Omo Valley Kibish Formation: Excavations, lithic assemblages, and inferred patterns of early Homo sapiens behavior. Journal of Human Evolution. 2008 Sep 1;55(3):448–85.

31. Clark JD, Beyene Y, WoldeGabriel G, Hart WK, Renne PR, Gilbert H, et al. Stratigraphic, chronological and behavioural contexts of Pleistocene Homo sapiens from Middle Awash, Ethiopia. Nature. 2003;423(6941):747.

32. Tryon CA, Roach NT, Logan MAV. The Middle Stone Age of the northern Kenyan Rift: age and context of new archaeological sites from the Kapedo Tuffs. Journal of Human Evolution. 2008 Oct 1;55(4):652–64.

33. Yellen J, Brooks A, Helgren D, Tappen M, Ambrose S, Bonnefille R, et al. The Archaeology of Aduma Middle Stone Age Sites in the Awash Valley, Ethiopia. PaleoAnthropology. 2005;10:25–100.

34. Yalçinkaya I. Thoughts on Levallois technique in Anatolia. In: The Definition and Interpretation of Levallois Technology. Madison, Wis. : Prehistory Press. Harold Lewis Dibble; Ofer Bar-Yosef; 1995. p. 399–412.

35. Otte M, Yalçinkaya I, Kozlowski J, Bar-Yosef O, López Bayón I, Taskiran H. Long-term technical evolution and human remains in the Anatolian Palaeolithic. Journal of Human Evolution. 1998 Apr 1;34(4):413–31.

36. Yaman ID. Orta Paleolitik Dönem’de Sedimantoloji ve Yontmataş Analizleri Işığında Karain Mağarası E ve B. Ankara University; 2013.

37. Baykara İ, Kuhn SL, Baykara DS. Mousterian lithic assemblages of Merdivenli cave. Mediterranean Archaeology and Archaeometry. 2016;16(1):101–15.

38. Panagopoulou E. The Theopetra Middle Palaeolithic assemblages: their relevance to the Middle Palaeolithic of Greece and adjacent areas. British School at Athens Studies. 1999;3:252–65.

39. Valladas H, Mercier N, Froget L, Joron J-L, Reyss J-L, Karkanas P, et al. TL age-estimates for the Middle Palaeolithic layers at Theopetra cave (Greece). Quaternary Geochronology. 2007 Jan 1;2(1):303–8.

40. Karkanas P, White D, Lane CS, Stringer C, Davies W, Cullen VL, et al. Tephra correlations and climatic events between the MIS6/5 transition and the beginning of MIS3 in Theopetra Cave, central Greece. Quaternary Science Reviews. 2015 Jun 15;118:170–81.

41. Guadelli J-L, Sirakov N, Ivanova S, Sirakova S, Anastassova E, Courtaud P, et al. Une séquence du paléolithique inférieur au paléolithique récent dans les Balkans : la grotte Kozarnika à Orechets (Nord-Ouest de la Bulgarie). Les Premiers Peuplements en Europe British Archaeological Reports, International Series. 2005;1364:87–103.

42. Tillier A, Sirakov N, Guadelli A, Fernandez P, Sirakova S, Dimitrova I, et al. Evidence of Neanderthals in the Balkans: The infant radius from Kozarnika Cave (Bulgaria). Journal of Human Evolution. 2017 Oct 1;111:54–62.

43. Dogandžić T, Đuričić L. Lithic production strategies in the Middle Paleolithic of the southern Balkans. Quaternary International. 2017 Sep 2;450:68–102.

44. Cyrek K, Czyzewski L, Sudoł-Procyk M. Middle Palaeolithic cultural levels from Middle and Late Pleistocene sediments of Biśnik Cave, Poland. Quaternary International. 2014;20–63.

45. Wiśniewski A. The beginnings and diversity of Levallois methods in the early Middle Palaeolithic of Central Europe. Quaternary International. 2014 Apr 1;364–80.

46. Picin A. Technological adaptation and the emergence of Levallois in Central Europe: new insight from the Markkleeberg and Zwochau open-air sites in Germany. Journal of Quaternary Science. 2018;33(3):300–12.

47. Van Baelen A, Meijs E PM, Van Peer P, De Warrimont J-P, De Bie M. An early Middle Palaeolithic site at Kesselt-Op de Schans (Belgian Limburg) Preliminary results. Notae Praehistoricae. 2007;27:19–26.

48. Van Baelen A, Meijs E PM, Van Peer P, De Warrimont J-P, De Bie M. The Early Middle Palaeolithic Site of Kesselt - Op de Schans (Belgian Limburg) Excavation Campaign 2008. Notae Praehistoricae. 2008;(28):5–9.

49. Cahen D. An Early Middle Palaeolithic Site at Mesvin IV (Mons, Belgium), Its Significance for Stratigraphy and Palaeontology. Institut royal des Sciences naturelles de Belgique. 1984. book.

50. Wenban-Smith F. Early Palaeolithic Cultural Facies and the Levalloisian at Baker’s Hole. Papers from the Institute of Archaeology. 1992 Nov 15;3(0):1–10.

51. Scott B, Ashton N. 7 - The Early Middle Palaeolithic: The European Context. In: Ashton N, Lewis SG, Stringer C, editors. Developments in Quaternary Sciences [Internet]. Elsevier; 2011. p. 91–112. (The Ancient Human Occupation of Britain; vol. 14). Available from: http://www.sciencedirect.com/science/article/pii/B9780444535979000078

52. Locht J-L, Goval E, Antoine P. Reconstructing Middle Palaeolithic hominid behaviour during OIS 5 in northern France. In: Settlement Dynamics of the Middle Paleolithic and Middle Stone Age [Internet]. Kerns Verlag Tübingen. Nicholas J. Conard, Anne Delagnes; 2010 [cited 2019 Apr 28]. Available from: https://www.researchgate.net/publication/260385808_Reconstructing_Middle_Palaeolithic_hominid_behaviour_during_OIS_5_in_northern_France

53. Locht J-L, Swinnen C, Antoine P, Révillion S, Depaepe P. Le gisement paléolithique moyen de Bettencourt-Saint-Ouen (Somme). In: L’Acheuléen dans la vallée de la Somme et le Paléolithique moyen dans le Nord de la France: données récentes [Internet]. Université des Sciences et Technologies de Lille: Alain Tuffreau; 2001 [cited 2019 Apr 28]. (Publications du CERP; vol. 6). Available from: https://www.researchgate.net/publication/324538262_Le_gisement_paleolithique_moyen_de_Bettencourt-Saint-Ouen_Somme

54. Locht J-L. Bettencourt-Saint-Ouen (Somme) Cinq occupations paléolithiques au début de la dernière glaciation [Internet]. Editions de la maison des Sciences de l’Homme. Paris; 2002 [cited 2019 Apr 28]. 169 p. (4; vol. 15). Available from: https://www.persee.fr/doc/quate_1142-2904_2004_num_15_4_1782_t1_0370_0000_2

55. Hérisson D, Locht J. Le niveau N3 de Therdonne : reflet de la diversité des technocomplexes du Paléolithique moyen ancien du Nord de la France ? In: Jaubert J, Fourment N, Depaepe P, editors. XXVIIe congrès préhistorique de France : transitions, ruptures et continuité en Préhistoire [Internet]. Bordeaux, France: Société Préhistorique Française; 2014. p. 43–58. (Paléolithique et Mésolithique; vol. Volume 2). Available from: https://hal.archives-ouvertes.fr/hal-01798521

56. Hérisson D. Étude des comportements des premiers Néandertaliens du Nord de la France. Les occupations saaliennes des gisements de Biache-Saint-Vaast et de Therdonne [Internet] [PhD Thesis]. Université Charles de Gaulle-Lille III; 2012 [cited 2015 Apr 19]. Available from: http://hal.univ-lille3.fr/tel-00824754/

57. Koehler H. L’apport du gisement des Osiers à Bapaume (Pas-de-Calais) au débat sur l’émergence du Paléolithique moyen dans le Nord de la France. Bulletin de la Société préhistorique française. 2008;105(4):709–35.

58. Delagnes A. Le site du Pucheuil à Saint-Saëns (Seine-Maritime): l’industrie lithique de la série B du Pucheuil. In: Delagnes A, Ropars A, editors. Paleolithique Moyen en Pays de Caux (Haute-Normandie): Le Pucheuil, Etoutteville, Deux Gisements de Plein-Air en Milieu Loessique. Paris: Éditions de la Maison des sciences de l’homme; 1996. p. 59–130.

59. Boëda E. Le concept Levallois: variabilité des méthodes. CNRS; 1994. 280 p.

60. Tuffreau A. Le gisement moustérien du château d’eau à Corbehem (Pas-de-Calais). Gallia Préhistoire. 1979;22(2):371–89.

61. Ameloot-Van der Heijden N. L’industrie laminaire du niveau C.A. du gisement paléolithique moyen de Riencourt-lès-Bapaume (Pas-de-Calais). Bulletin de la Société préhistorique française. 1993;90(5):324–7.

62. Moncel M, Daujeard C. The variability of the Middle Palaeolithic on the right bank of the Middle Rhône Valley (southeast France): Technical traditions or functional choices? Quaternary International. 2012 Jan 9;247:103–24.

63. Mathias C. Les phases anciennes du Paléolithique moyen dans le Sud-Est et le Sud-Ouest de la France : étude des systèmes techniques lithiques [Internet] [PhD Thesis]. Université de Perpignan; 2018 [cited 2019 Feb 11]. Available from: https://tel.archives-ouvertes.fr/tel-01960322/document

64. Notter O. Etudes des industries lithiques du paléolithique inférieur et moyen de la grotte de la Baume Bonne (Quinson, Alpes de Haute-Provence, France) [Internet] [thesis]. Aix-Marseille 1; 2007 [cited 2019 Apr 28]. Available from: http://www.theses.fr/2007AIX11075

65. Moncel M-H. Baume Flandin et Abri du Maras : deux exemples de débitage laminaire du début du Pléistocène supérieur dans la Vallée du Rhône (sud-est, France). L’Anthropologie. 2005;3(109):451–80.

66. Defleur A. Les industries lithiques moustériennes de la Baume Moula-Guercy (Soyons, Ardèche). Fouilles 1993–1999. L’Anthropologie. 2015 Apr 1;119(2):170–253.

67. Defleur AR, Desclaux E. Impact of the last interglacial climate change on ecosystems and Neanderthals behavior at Baume Moula-Guercy, Ardèche, France. Journal of Archaeological Science. 2019 Apr 1;104:114–24.

68. Carmignani L, Moncel M-H, Fernandes P, Wilson L. Technological variability during the Early Middle Palaeolithic in Western Europe. Reduction systems and predetermined products at the Bau de l’Aubesier and Payre (South-East France). PLOS ONE. 2017 juin;12(6):e0178550.

69. Brenet M. Variabilité et signification des productions lithiques au Paléolithique moyen ancien : l’exemple de trois gisements de plein-air du Bergeracois (Dordogne, France) [Internet] [thesis]. Bordeaux 1; 2011 [cited 2019 Apr 28]. Available from: http://www.theses.fr/2011BOR14356

70. Frouin M, Lahaye C, Hernandez M, Mercier N, Guibert P, Brenet M, et al. Chronology of the Middle Palaeolithic open-air site of Combe Brune 2 (Dordogne, France): a multi luminescence dating approach. Journal of Archaeological Science. 2014 Dec 1;52:524–34.

71. Brenet M, Bourguignon L, Colonge D, Folgado M, Jarry M, Lelouvier L-A, et al. Les techno-complexes au début du Paléolithique moyen en Aquitaine septentrionale : complexité, complémentarité des productions de débitage et de façonnage et implications comportementales. In: Jaubert, Jacques, Fourment, Nathalie, Depaepe, P., editors. Transitions, Ruptures et Continuité en Préhistoire : XXVIIème Congrès Préhistorique de France, Bordeaux-Les Eyzies 31 mai-5 juin 2010 [Internet]. Bordeaux-Les Eyzies, France: Paris : Société Préhistorique Française; 2013. p. 81–101. (Paléolithique et Mésolithique; vol. 2). Available from: https://hal.archives-ouvertes.fr/hal-01836340

72. Rigaud J-P. La grotte Vaufrey, Dordogne [Internet]. Société Préhistorique Française; 1988 [cited 2019 Apr 28]. 616 p. Available from: http://www.prehistoire.org/shop_515-17340-2674-822/m19-1988-la-grotte-vaufrey-dordogne-j.-p.-rigaud.html

73. Guibert P, Bechtel F, Bourguignon L, Brenet M, Couchoud I, Delagnes A, et al. Une base de données pour la chronologie du paléolithique moyen dans le Sud-Ouest de la France. 2008;19–40.

74. Jaubert J. Le site moustérien du Rescoundudou (Sébazac-Concourès, Aveyron), présentation et problématique. Bulletin de la Société préhistorique française. 1983;80(3):80–7.

75. Jaubert J, Mourre V. Coudoulous, Le Rescoundudou, Mauran : diversité des matières premières et variabilité des schémas de production d’éclats. Quaternaria Nova VI. 1996;Bietti, A. et Grimaldi, S. Reduction processes for the European Mousterian, Proceedings of the International Round Table, Rome:313–41.

76. Jaubert J, Kervazo B, Mourre V, Bahain J-J, Brugal J-P, Chalard P, et al. Coudoulous I (Tour-de-Faure, Lot), site du Pléistocène moyen en Quercy: Bilan pluridisciplinaire. British Archaeological Reports (BAR Int Series) [Internet]. 2004 [cited 2019 Apr 28]; Available from: https://www.academia.edu/1197899/Coudoulous_I_Tour-de-Faure_Lot_site_du_Pl%C3%A9istoc%C3%A8ne_moyen_en_Quercy_Bilan_pluridisciplinaire

77. Delagnes A, Tournepiche J-F, Armand D, Desclaux E, Diot M-F, Ferrier C, et al. Le gisement Pléistocène moyen et supérieur d’Artenac (Saint-Mary, Charente) : premier bilan interdisciplinaire. Bulletin de la Société préhistorique française. 1999;96(4):469–96.

78. Armand D. La faune de la grotte Bourgeois-Delaunay(commune de La Chaise de Vouthon, Charente). Résultats préliminaires/The fauna from the grotte de Bourgeois-Delaunay (Chaise de Vouthon, Charente). Preliminary results. Paléo, Revue d’Archéologie Préhistorique. 1998;10(1):77–86.

79. Delagnes A, Rendu W. Shifts in Neandertal mobility, technology and subsistence strategies in western France. Journal of Archaeological Science. 2011 Aug 1;38(8):1771–83.

80. Meignen L. L’abri des Canalettes : un habitat moustérien sur les grands Causses (Nant, Aveyron) : fouilles 1980-1986 [Internet]. Monographie du CRA 10. CNRS; 1993 [cited 2019 Feb 11]. Available from: https://gallica.bnf.fr/ark:/12148/bpt6k3333276w

81. Turq A. Le paléolithique inférieur et moyen entre Dordogne et Lot. Les Eyzies de Tayac-Sireuil: Société des amis du Musée national de Préhistoire et de la recherche archéologique; 2000. 456 p.

82. Bourguignon L, Blaser F, Rios J, Pradet L, Sellami F, Guibert P. L’occupation moustérienne de la Doline de Cantalouette II (Creysse, Dordogne): spécificités technologiques et économiques, premiers résultats d’une analyse intégrée. In: Les sociétés du Paléolithique dans un Grand Sud-Ouest de la France Nouveaux gisements, nouveaux résultats, nouvelles méthodes Journées SPF,. Société Préhistorique Française. Paris; 2008. p. 133–50.

83. Kervazo B, Turq A, Diot M-F. Le site Moustérien de plein air de la Plane, commune de Mazeyrolles, Dordogne : note préliminaire. Bulletin de la Société préhistorique française. 1989;86(9):268–74.

84. Collina-Girard J, Turq A. Le Paléolithique moyen sur galets de la station des Planes, commune de Montayral (Lot-et-Garonne). Paléo, Revue d’Archéologie Préhistorique. 1991;3(1):49–74.

85. Picin A, Peresani M, Falguères C, Gruppioni G, Bahain J-J. San Bernardino Cave (Italy) and the Appearance of Levallois Technology in Europe: Results of a Radiometric and Technological Reassessment. PLOS ONE. 2013 Oct 16;8(10):e76182.

86. Soriano S, Villa P. Early Levallois and the beginning of the Middle Paleolithic in central Italy. PLOS ONE. 2017 Oct 20;12(10):e0186082.

87. Eixea A. Caracterización tecnológica y uso del espacio en los yacimientos del Paleolítico medio de la región central del Mediterráneo Ibérico [Internet] [PhD Thesis]. Universitat de Valencia; 2015 [cited 2019 Apr 27]. Available from: http://mobiroderic.uv.es/handle/10550/47842

88. Eixea A, Villaverde V, Zilhão J. Not Only Flint: Levallois on Quartzite and Limestone at Abrigo de la Quebrada (Valencia, Spain): Implications for Neandertal Behavior. Journal of Anthropological Research. 2016 Mar 1;72(1):24–57.

89. Eixea A. Middle palaeolithic lithic assemblages in western Mediterranean Europe from MIS 5 to 3. Journal of Archaeological Science: Reports. 2018 Oct 1;21:643–66.
