# Supplementary figures and images for: New insights into early MIS 5 lithic technological behavior in the Levant: Nesher Ramla, Israel as a case study
Source: PLoS One. 2020 Apr 3;15(4):e0231109. doi: 10.1371/journal.pone.0231109 (PMC7122790; doi:10.1371/journal.pone.0231109)

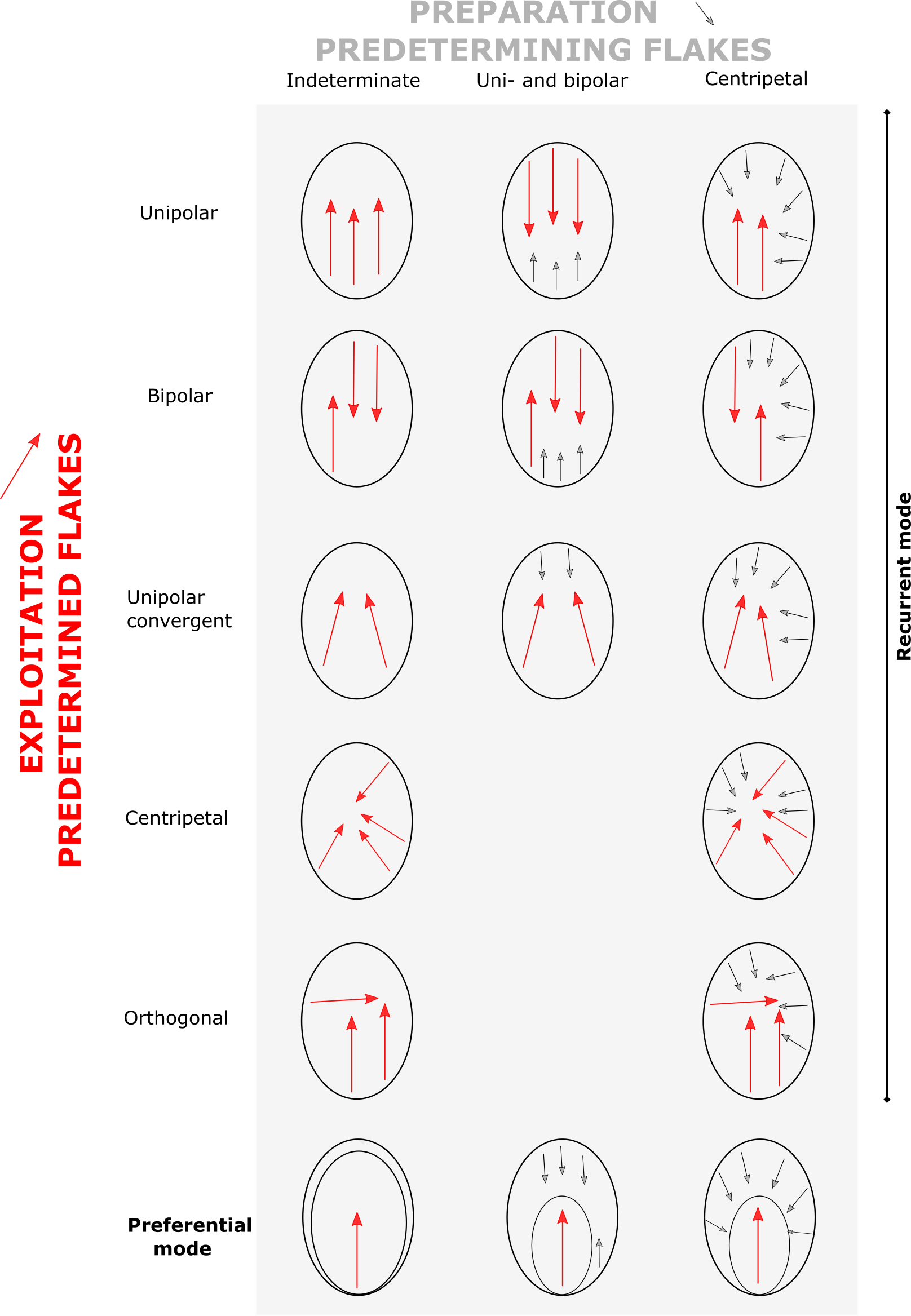


S1 Fig. Levallois systems according to different modes, methods, exploitation and preparation.

Supplement: S1 Fig — (DOCX) [file pone.0231109.s006.docx]
