# Supplementary material for: New insights into early MIS 5 lithic technological behavior in the Levant: Nesher Ramla, Israel as a case study
Source: PLoS One. 2020 Apr 3;15(4):e0231109. doi: 10.1371/journal.pone.0231109 (PMC7122790; doi:10.1371/journal.pone.0231109)
Supplement: S2 Fig — Yellow color illustrates last additional flake removal. Grey color illustrates preparation flakes (predetermining). (DOCX) [file pone.0231109.s007.docx]

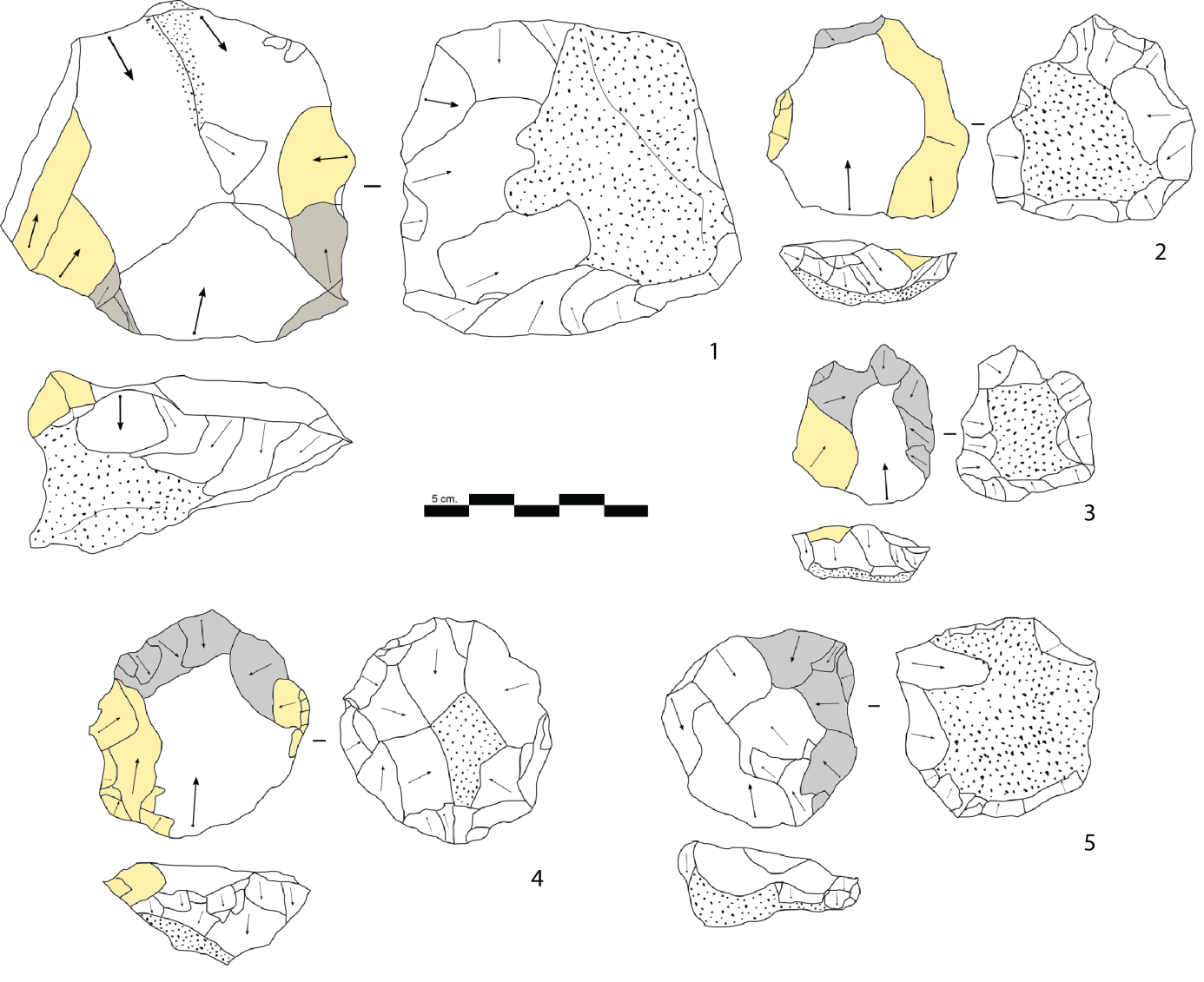


S2 Fig. Levallois cores. Yellow color illustrates last additional flake removal. Grey color illustrates preparation flakes (predetermining).
