# Supplementary material for: New insights into early MIS 5 lithic technological behavior in the Levant: Nesher Ramla, Israel as a case study
Source: PLoS One. 2020 Apr 3;15(4):e0231109. doi: 10.1371/journal.pone.0231109 (PMC7122790; doi:10.1371/journal.pone.0231109)
Supplement: S3 Fig — 1. Pseudo-Levallois points/flakes can generate different scar patterns organization according to their place on the surface. 2. Position of a débordant flake and schematized scar pattern. (DOCX) [file pone.0231109.s008.docx]

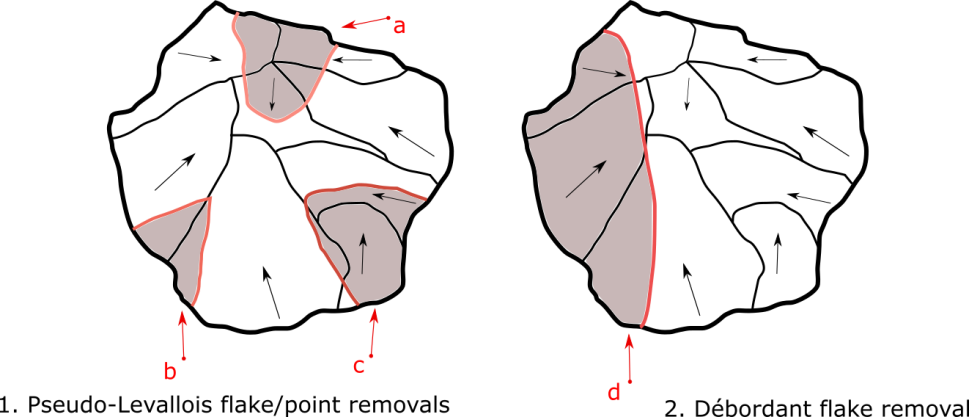


S3 Fig. Schematic illustrations of pseudo-Levallois/points removals and débordant flake removal on a centripetal Levallois core flaking surface. 1. Pseudo-Levallois points/flakes can generate different scar patterns organization according to their place on the surface. 2. Position of a débordant flake and schematized scar pattern.
